# Recruiting foreign-born individuals who have sought an abortion in the United States: Lessons from a feasibility study

**DOI:** 10.3389/fgwh.2023.1114820

**Published:** 2023-04-18

**Authors:** Carmela Zuniga, Sachiko Ragosta, Terri-Ann Thompson

**Affiliations:** ^1^ Ibis Reproductive Health, Cambridge, MA, United States; ^2^ Ibis Reproductive Health, Oakland, CA, United States

**Keywords:** abortion, United States, immigrants, recruitment, hard-to-reach population

## Abstract

Although studies have documented challenges people encounter when attempting to access abortion care in the United States, there is little research on the perspectives and experiences of foreign-born individuals, who may encounter unique barriers to accessing care. Since lack of data may be due to difficulty recruiting this population, we explored the feasibility of using social media to recruit foreign-born individuals who have sought an abortion into interviews to share their abortion experiences. Our target population was limited to English and Spanish-speakers due to budget constraints. As this recruitment method was unsuccessful, we attempted to recruit our target population through the crowdsourcing website, Amazon Mechanical Turk (mTurk) to take a one-time survey on their abortion experience. Both online recruitment methods yielded a significant number of fraudulent responses. Although we aimed to collaborate with organizations that work closely with immigrant populations, they were unavailable to assist with recruitment efforts at the time of the study. Future abortion research utilizing online methods to recruit foreign-born populations should consider incorporating information on their target populations' use of online platforms as well as cultural views on abortion in order to develop effective recruitment strategies.

## Introduction

Little data exist on the demographics and experiences of foreign-born people (anyone not a US citizen at birth) (1) seeking or receiving abortions in the US. One study exploring national patient survey data (gathered in 2008–2009 and 2013–2014) to understand the characteristics of immigrants who had an abortion found that the majority were in their 20s, earned poverty-level or near poverty-level incomes, and had graduated from college or had some college education (2). This study also found that compared with US-born women, a greater proportion of immigrant women were older, did not have health insurance, and did not graduate from high school.

Foreign-born individuals make up a diverse population in the United States, and previous literature suggests that their experiences accessing abortion may differ by factors such as their country of origin, level of income, and state or city of residence. One study using surveillance data to examine the use of abortions services among different Asian groups in New York City from 2011 to 2015 found that abortion rates were higher for US-born Asian groups compared to foreign-born groups, and analysis by country of origin showed that abortion rates were the highest among Indian women (30.5 abortions per 1,000 women) and lowest among Korean women (5.1 abortions per 1,000 women) (3). Another study exploring barriers to abortion access among Mexican immigrants residing in North Carolina described how abortions services are restricted by legal and medical institutions, and may have led some to seek their abortion outside of a formal healthcare setting (4). Other data on abortion among foreign-born individuals come from smaller studies conducted over a decade ago on people earning low-incomes in certain locations (San Francisco, Boston, New York City, Massachusetts), and highlight lack of knowledge about abortion laws (5) and difficulties navigating the health care system as barriers (5, 6). However, no studies explore barriers or facilitators foreign-born individuals encounter at a national level when attempting to access abortion services.

Immigration status has been shown to be an influencing factor on access to health services, so it may also impact one's abortion seeking experience. Some foreign-born individuals reside in the US temporarily (such as students, temporary workers, and tourists), whereas others intend to live in the US permanently, including individuals with immigrant visas, individuals who have been lawfully authorized to live in the US permanently (known as lawful permanent residents or green card holders), naturalized citizens, and individuals residing in the US without authorization (7, 8). For foreign-born individuals attempting to become lawfully permanent residents, fear of being deemed a “public charge”^1^ for using certain public programs or services (even if they are eligible for these programs and services) has led some to avoid publicly-funded programs (9) and may be a barrier to accessing public health insurance or using of health services, including abortion care. In addition, undocumented immigrants, are more likely to be uninsured (10, 11) and have lower incomes (10) than US-born individuals, so cost may be a common barrier to seeking abortion care at a facility for this population (9–12).

The dearth of demographic data and data on abortion seeking experiences among foreign-born individuals may, in part, be due to difficulty recruiting this population, particularly those who are undocumented or who have an undocumented relative. These individuals may be reluctant to participate in research studies due to fear that any information they provide could be shared with authorities and result in deportation (9), making them especially difficult to recruit into studies. Abortion stigma may also contribute to foreign-born individuals being difficult to recruit into a study about abortion. People who have sought or have had an abortion may fear being judged by others or may feel shame themselves, so many individuals may choose to keep their abortion or abortion seeking experience a secret (13). Some foreign-born individuals may experience abortion stigma more acutely than others due to their culture, country of origin, length of time in the US, age, and other factors.

Studies have successfully used social media (14–16), peer referral (14, 17–21), and referrals by trusted organizations or facilities (14, 17–21) to recruit hard-to-reach populations. Some studies using multiple recruitment methods found that referral through trusted organizations and/or peers to be the most effective (14, 18, 19), and others noted that social media recruitment was faster (15), less expensive (15, 17), or had a wider geographical reach (17) than other methods. Among studies that have recruited foreign-born individuals in particular, peer referral as well as collaboration with well-respected institutions, organizations, or individuals, were effective recruitment tools (18–21). Given our hypothesis that foreign-born individuals who have sought an abortion would be challenging to recruit, we aimed to conduct a pilot study to assess the feasibility of recruiting a small, diverse sample of this population using a mix of trusted and/or cost-effective techniques.

## Materials and methods

### Eligibility criteria

Individuals were eligible to participate if they were: born outside the US, between the ages of 12–49 years, lived in the US at the time of the study, sought an abortion in the US in the past 2 years (regardless of whether they received an abortion or not), and spoke English or Spanish. Since this was a small pilot study with a limited budget, we were unable to conduct the study in more than two languages. Spanish and English were chosen since they are the most common languages spoken by immigrants, with 42% of immigrants reporting they speak Spanish at home and 17% reporting they only speak English at home (22).

### Recruitment

We aimed to recruit and interview 30 foreign-born individuals from across the country (23). We planned to share online posts on a variety of social media platforms and conduct online recruitment with the support of organizations that work closely with foreign-born individuals. In the fall of 2020, we reached out to eleven organizations that work closely with foreign-born populations to assess their capacity to assist with recruitment efforts, including by sharing study information through: flyers, social media posts, and/or relevant listservs. We planned to supplement our online recruitment efforts with snowball sampling by asking participants at the end of their interview to share this study with other foreign-born individuals they knew who had tried to get an abortion in the US.

Online posts (Supplementary Appendix S1) directed potential participants to a study webpage (Supplementary Appendix S2), which described the study objective and linked to a short online screening questionnaire (Supplementary Appendix S3). Eligible participants provided electronic consent to participate in the study, completed a short demographic survey, and provided contact information for scheduling a Zoom interview (audio only). All electronic survey data were collected through Qualtrics. The interview guide (Supplementary Appendix S4) explored the following topics: the number of times participants desired an abortion and the number of abortions obtained in the United States; participants' decision-making process that led them to seek an abortion for their most recent pregnancy; participants' abortion seeking experience, and experiences obtaining an abortion or reasons for not obtaining one. The guide also included questions about participants' use (or non-use) of contraception while in the United States. Those completing an interview received a $40 gift card.

In our conversations with individuals that conducted research with or worked closely with foreign-born individuals and communities, two recommendations for improving our study design emerged. The first was for us to provide participants with a list of relevant health-related resources, such as mental health services, in addition to a gift card since the act of sharing their abortion experience may be traumatic for some participants. They also suggested we limit recruitment efforts to certain states or cities with large foreign-born populations instead of recruiting nation-wide for the pilot study to increase our chances of recruiting our target population. In response, we created a resource guide (an English and a Spanish version) with a list of national and local organizations providing: counseling for abortion and pregnancy-related experiences, information on abortion law and self-managed abortion, legal assistance, financial and logistical support for abortion care, mental health services, and other health-related resources. We refined our resource guides to focus on 7 cities across the US with large immigrant populations: Atlanta, Boston, Chicago, Los Angeles, New York, San Francisco, and Saint Paul and Minneapolis (see Supplementary Appendix S5 for an examples of one of these guides).

This study was approved by Allendale Investigational Review Board.

## Results

We reached out to two organizations that were nationally-focused and nine organizations that worked in specific states. Three worked with immigrants identifying as Latinx, two worked with Black immigrants, and six worked with foreign-born populations broadly. One organization responded and informed us they could not assist with recruitment efforts because they were “swamped”. However, this organization was willing to offer recommendations about our study design and implementation. We also sought and received advice about our study design from a researcher who has experience working with immigrant populations. All other organizations we attempted to contact did not respond. Therefore, we relied solely on our research organization's social media platforms. We advertised in the form of organic posts in English and Spanish to our research organization's Facebook, Twitter, and LinkedIn accounts from March 16–31 2021. These posts, as they were on our research organization's accounts, could not be targeted to certain states or populations, but instead were visible to followers of our research organization's accounts and networks. Analytic data reveal that posts received 574 views and one click on Facebook, and 6,200 views and 11 clicks on Twitter. Data on the number of views and clicks were not available for LinkedIn.

During the month of March 2021, 6 screening questionnaires were completed, and three participants were eligible for interviews. However, closer inspection of Internet Protocol (IP) addresses and location (latitude and longitude) data revealed that one participant had completed the screener multiple times. In June, we received 12 additional screening questionnaires, and 11 were completed on devices with the same or similar IP addresses, or from the same location. Only one questionnaire was completed by a different person, but that individual was ineligible to participate.

### Shifts in recruitment and data collection strategy

Given the slow recruitment pace and challenges with duplicate responses, we modified our recruitment and data collection strategies. We recruited through Amazon's Mechanical Turk (mTurk) – an online platform in which workers are paid to complete tasks – since it has been widely used by researchers, has a large pool of workers based in the US (24), and is cost effective (25). The demographics of US-based mTurk workers also seemed promising for recruiting our target population – mTurk workers tended to be younger and earn lower incomes than the general US population (24) (as stated above, the majority of foreign-born individuals who had an abortion are in their 20 s and earn low incomes). In addition, over half of mTurk workers identify as women (26).^2^ mTurk workers also have an approval rating that reflects the quality of work they have previously completed. If work is of low-quality, researchers can reject the submission, which lowers the worker's approval rating and results in the worker being unpaid, which we thought would deter people from submitting fraudulent responses. mTurk also uses device fingerprinting and conducts network location analysis to validate mTurk workers' identities, and US workers are required to complete a tax interview and provide official forms of identification (27).

To better align our data collection strategy with mTurk's platform, we first converted the interview guide questions into survey questions and added the demographic questionnaire to the end of the survey (Supplementary Appendix S6). Additionally, we narrowed the survey to focus on abortion experiences only. Finally, we adjusted our eligibility criteria to only include foreign-born individuals aged 18–49 years (since mTurk workers cannot be under the age of 18), identifying as female, and who had a high (90%) approval rating for previously completed mTurk tasks.

We posted a screening questionnaire as a “task” on mTurk in both English and Spanish (Supplementary Appendix S7). The estimated time to take the questionnaire was one minute or less and workers received $0.20, which is double the minimum rate many workers consider fair (28). All participants who completed the screening questionnaire received compensation through mTurk. Responses were then reviewed by the study team and eligible participants were able to view a link to the online survey. Upon completing the online survey, responses were reviewed and if approved by the study team, a payment of $10 was sent through mTurk. At the end of the survey, participants were informed that resource guides containing information about health-related services were available on the study webpage. We aimed to collect data from 50 to 75 participants, with an equal distribution of survey responses in English and Spanish.

### Results from mTurk recruitment

Between October 20, 2021–January 28, 2022, 900 people completed the eligibility screener; of whom 164 were eligible. One-hundred and six surveys were completed in English and two in Spanish. One-hundred and three responses were deemed fraudulent due to one or more of the following reasons: duplicate IP addresses, responses that had contradictions or were otherwise infeasible, and suspicious or nonsensical responses (for example, reporting an earlier gestational age at the time of the abortion than when they found out they were pregnant, or reporting a fewer number of pregnancies than abortions). Table 1 displays recruitment metrics.

**Table 1:** Online recruitment metrics.

Number screened	Number deemed eligible	Number actually eligible	Recruitment rate (Number actually eligible/number screened)
**Social Media Recruitment**			
6	3	0	0/6 (0%)
12	0	0	0/12 (0%)
**Mturk Recruitment**			
900	164	5	5/900 (1%)

We also encountered difficulties recruiting Spanish-speakers, as almost all survey responses were in English – even by people who filled out the Spanish screener. We edited the instructions to clarify that only people completing surveys in Spanish would receive compensation, but continued receiving responses in English.

## Discussion

Recruiting foreign-born individuals who have sought an abortion in the US proved ineffective through social media and mTurk. Recruiting through social media channels yielded a significant amount of fraudulent responses and one possible reason for this was the incentive amount ($40). One study found that incentives between $15–30 increased the likelihood of a participant submitting multiple responses as much as six times, compared to unpaid participants (29). Recruiting on mTurk also resulted in numerous fraudulent responses despite reducing compensation from $40 to $10. Additionally, having an approval rating that reflects mTurk workers' quality of work did not deter workers from submitting fraudulent responses. It seems that very few researchers reject any submissions due to IRB restrictions or desire to compensate all workers for their time, which may contribute to approval ratings that do not accurate reflect quality of work as well as to the high frequency of fraudulent responses (30).

We were unable to partner with trusted community organizations for recruitment, which studies have noted as key to recruiting hard-to-reach populations (31, 32). As such, we were limited to reaching individuals familiar with our organization and their peers. We were also limited to recruiting English and Spanish speakers, so eligible individuals that did not speak either of these languages were excluded.

We reached out to organizations in the fall, right before the 2020 US presidential elections, which may explain why organizations could not assist with recruitment efforts. In addition, our recruitment period coincided with the COVID-19 pandemic, which has disproportionally impacted immigrants. Immigrants, particularly women, encountered higher unemployment rates than US-born workers from March 2020–June 2021 (33), and many continued to work in essential industries, such as food services or health care, putting them and their families at higher risk of COVID-19 exposure (34). This backdrop of current events most likely made our target population even more difficult to recruit (31–34).

Although recruiting through mTurk yielded more eligible participants than social media, the number of eligible participants was extremely low even though some demographic characteristics of mTurk workers (such as age and income) seemed promising for recruiting our target population. mTurk may not have been the best recruitment platform for this population because of other characteristics, like race, ethnicity, and immigration status. Research suggests that Hispanics make up the largest proportion of foreign-born individuals obtaining abortions (2) yet Hispanics of all races are under-represented among mTurk workers (24). Undocumented immigrants may also have been under-represented, as they may lack the necessary tax documents or forms of identification that mTurk requires for employment. It is possible that certain characteristics, such as country of origin or age, impact use of online platforms. One study on strategies for recruiting Brazilian immigrants found that Facebook was an effective method for recruiting first-time pregnant women, while in-person outreach and snowball sampling was the most effective method for recruiting Brazilian immigrant parents (35). Another study examining correlates of cervical cancer screening behaviors among African immigrant women in the US found WhatsApp to be an effective peer recruitment platform, and noted that the suggestion to use this particular platform came from key informants, well respected by and knowledgeable about their target population (19). Studies that have recruited immigrants and minorities have noted the importance of understanding a target populations' culture (19, 21, 36). Our recruitment rates may have been greatly improved had we narrowed the study to focus on specific foreign-born communities and taken into consideration those communities' use of online platforms.

Lastly, the sensitive and often stigmatized topic of abortion itself may have deterred individuals from participating in the study, and views about abortion may vary by country of origin, race and/or ethnicity, and other factors. Research reveals that compared to US-born individuals, foreign-born Hispanic and Black individuals are more likely to perceive abortion stigma among their friends or family and keep their abortion a secret.(37) Another study among a sample of Asian Americans (38% of which were foreign-born) found that abortion stigma prevented most participants from disclosing their abortion to family members and led to feelings of isolation throughout their abortion experience (38). As abortion stigma influences language about abortion, engaging with trusted members of the community can ensure that researchers use abortion terminology in study materials that members of the target population would likely feel the most comfortable with (39).

Understanding challenges encountered by foreign-born individuals seeking abortion care is critical to addressing their needs – especially as the *Dobbs v Jackson* decision overturned the constitutional right to abortion, allowing states to severely restrict or ban abortion care (40). While we were unable to assess whether engagement with trusted community organizations was a successful or unsuccessful recruitment strategy, we found recruiting foreign-born individuals through social media without community partners and mTurk to be ineffective. Future abortion research focusing on recruiting foreign-born populations online should consider identifying specific sub-groups of this population and incorporating information on their target populations' use of online platforms as well as cultural views on abortion into their efforts. Trusted community organizations will be an important part of the research process to become well-informed about the target population and develop appropriate recruitment strategies.

## Data Availability

The raw data supporting the conclusions of this article will be made available by the authors, without undue reservation.

## Footnotes

^1^Non-citizens that are (or are likely to be) dependent on certain publicly funded programs are considered a public charge, which makes them ineligible to obtain lawful permanent residence. https://www.dhs.gov/news/2022/09/08/dhs-publishes-fair-and-humane-public-charge-rule#:∼:text=A%20noncitizen%20who%20is%20deemed,colloquially%20as%20a%20green%20card).

^2^mTurk workers are given the option to identify as men or women, and these two categories may not accurately represent the identities of trans and gender-expansive people; the term women in this context may only refer to cisgender women.
