# Supplementary material for: Recruiting foreign-born individuals who have sought an abortion in the United States: Lessons from a feasibility study
Source: Front Glob Womens Health. 2023 Apr 18;4:1114820. doi: 10.3389/fgwh.2023.1114820 (PMC10151930; doi:10.3389/fgwh.2023.1114820)
Supplement: Supplementary file 1 [file Datasheet1.zip › Appendix 1.PDF]

Social Media Text (English)

Social Media Messages (either posted alone or with graphics, depending on social media platform)

1. Were you born outside the US? We want to hear about your experience seeking abortion care in the US. Share your experience with us and receive a $40 gift card!

   Learn more here: [study URL] #AbortionAccess #immigranthealth #SRH #ReproductiveFreedom #ReproductiveHealth

2. Want to help improve #AbortionAccess in the US? We're looking for foreign-born individuals who have tried to get an abortion in the US to share their experience with us.

Learn more here: [study URL] #AbortionAccess #ImmigrantHealth #SRH #ReproductiveFreedom #ReproductiveHealth

Social Media Text (Spanish)

1. ¿Nació fuera de los Estados Unidos? Queremos escuchar su experiencia en la búsqueda de servicios de aborto seguro en los EE. UU. ¡Comparta su experiencia con nosotros y reciba una tarjeta de regalo de $ 40!

   Para más información, haga clic aquí: [study URL] #AccesoAlAborto #AbortionAccess #SaludDeInmigrantes #ImmigrantHealth #SSR #SRH #LibertadReproductiva #ReproductiveFreedom #SaludReproductiva #ReproductiveHealth

2. ¿Quiere ayudar a mejorar el #AccesoAlAborto #AbortionAccess en los Estados Unidos? Estamos buscando personas nacidas en el extranjero que hayan intentado abortar en los Estados Unidos para compartir su experiencia con nosotrxs.

   Aprenda más aquí: [study URL] #AccesoAlAborto #AbortionAccess #SaludDeInmigrantes #ImmigrantHealth

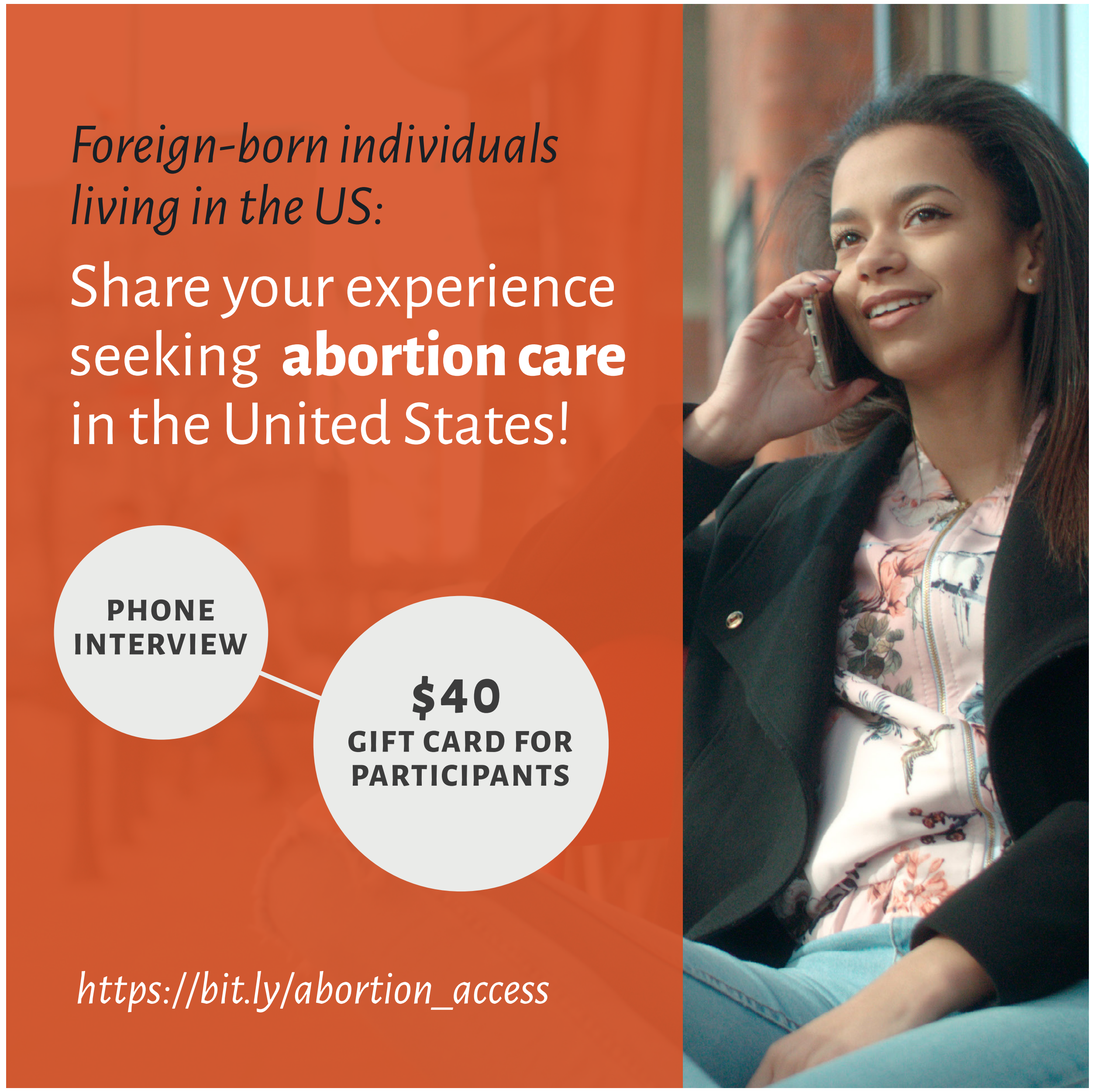
Foreign-born individuals living in the US:
Share your experience seeking **abortion care** in the United States!
PHONE INTERVIEW
$40
GIFT CARD FOR PARTICIPANTS
https://bit.ly/abortion_access

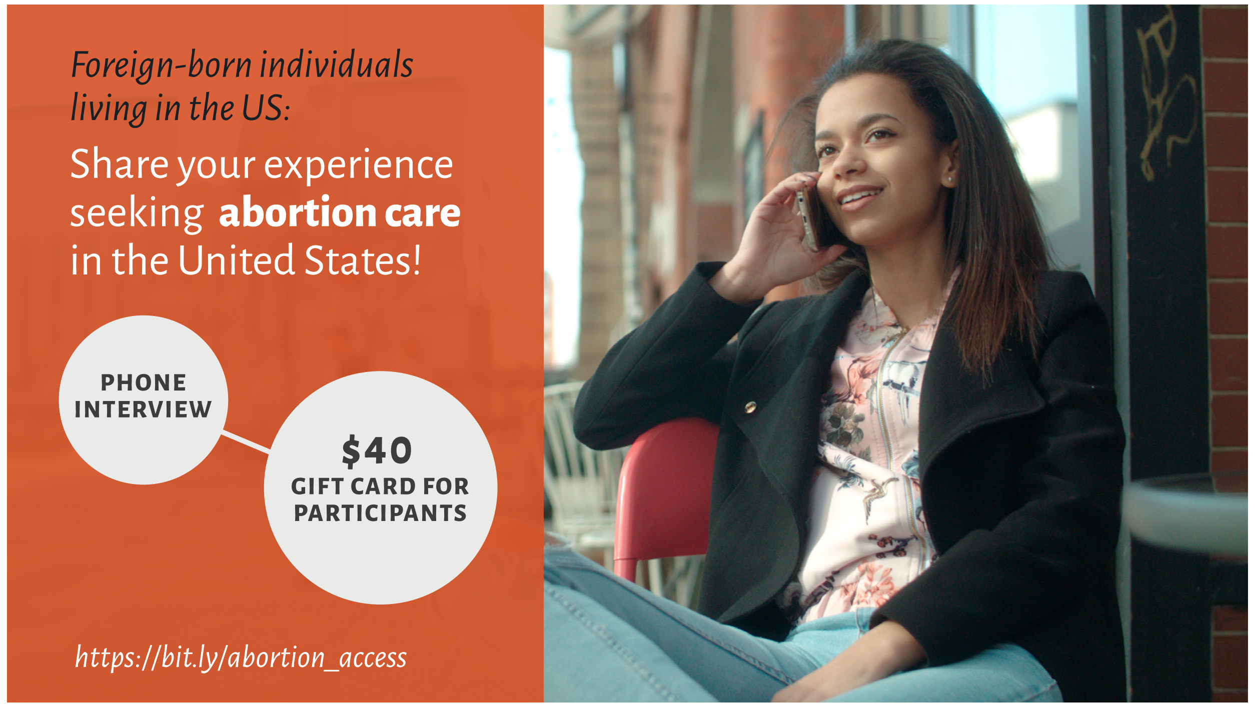
Foreign-born individuals living in the US:
Share your experience seeking **abortion care** in the United States!
PHONE INTERVIEW
$40
GIFT CARD FOR PARTICIPANTS
https://bit.ly/abortion_access

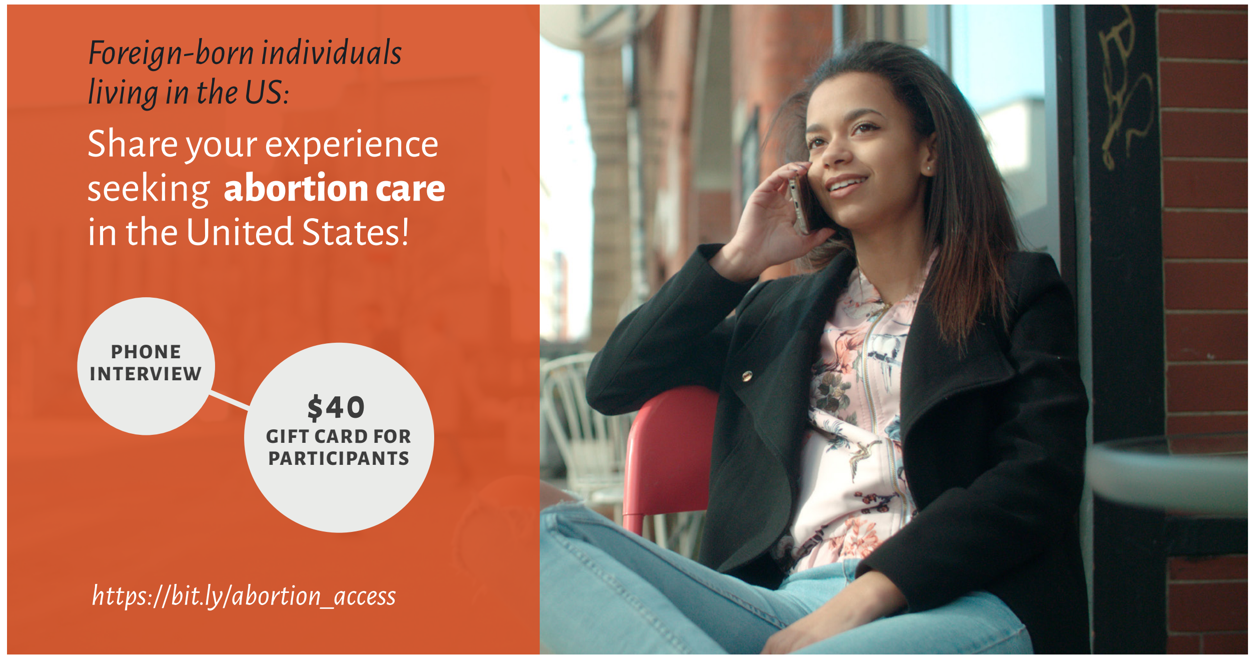
Foreign-born individuals living in the US:
Share your experience seeking **abortion care** in the United States!
PHONE INTERVIEW
$40
GIFT CARD FOR PARTICIPANTS
https://bit.ly/abortion_access

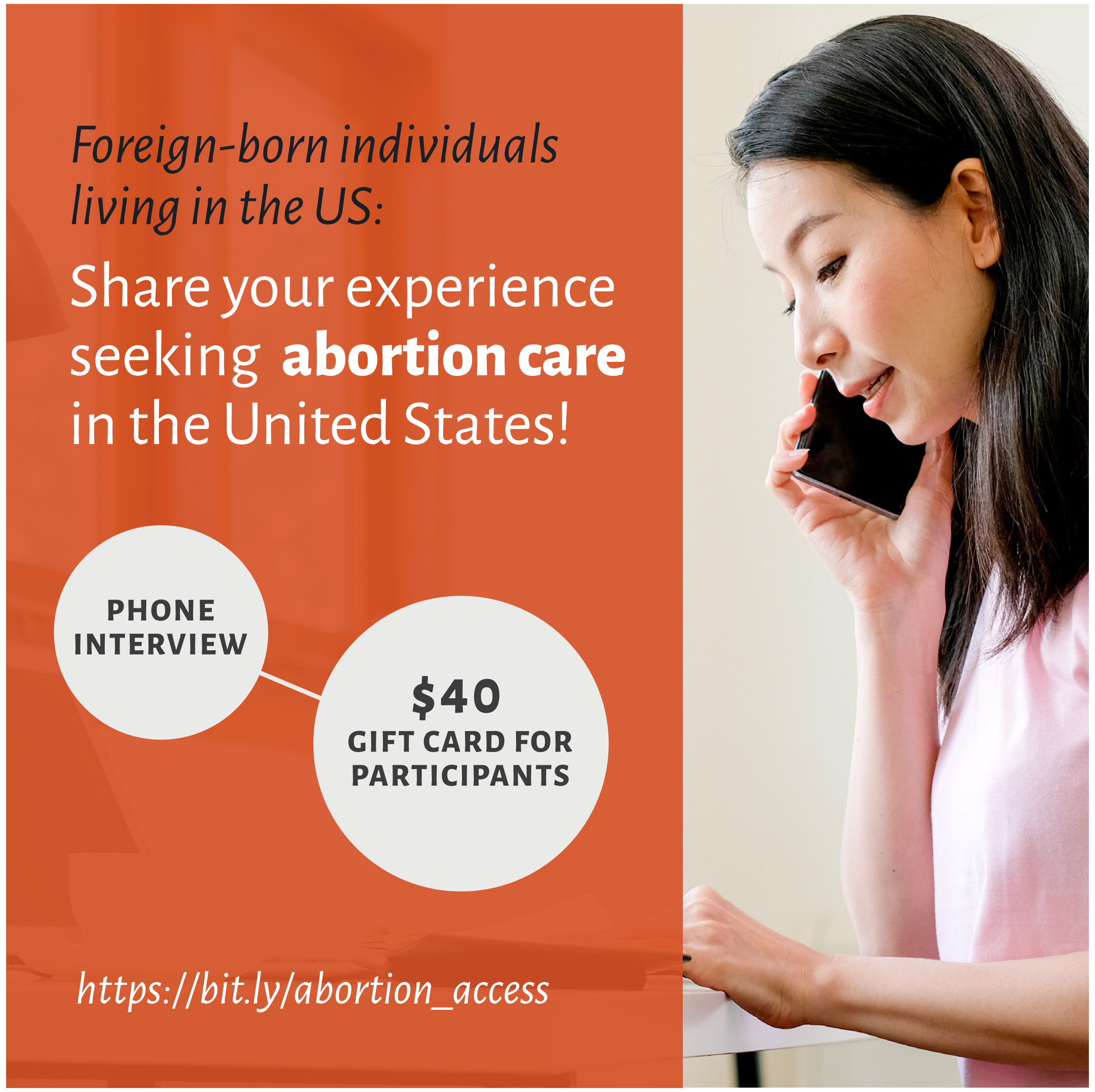
Foreign-born individuals living in the US:
Share your experience seeking **abortion care** in the United States!
PHONE INTERVIEW
$40 GIFT CARD FOR PARTICIPANTS
https://bit.ly/abortion_access

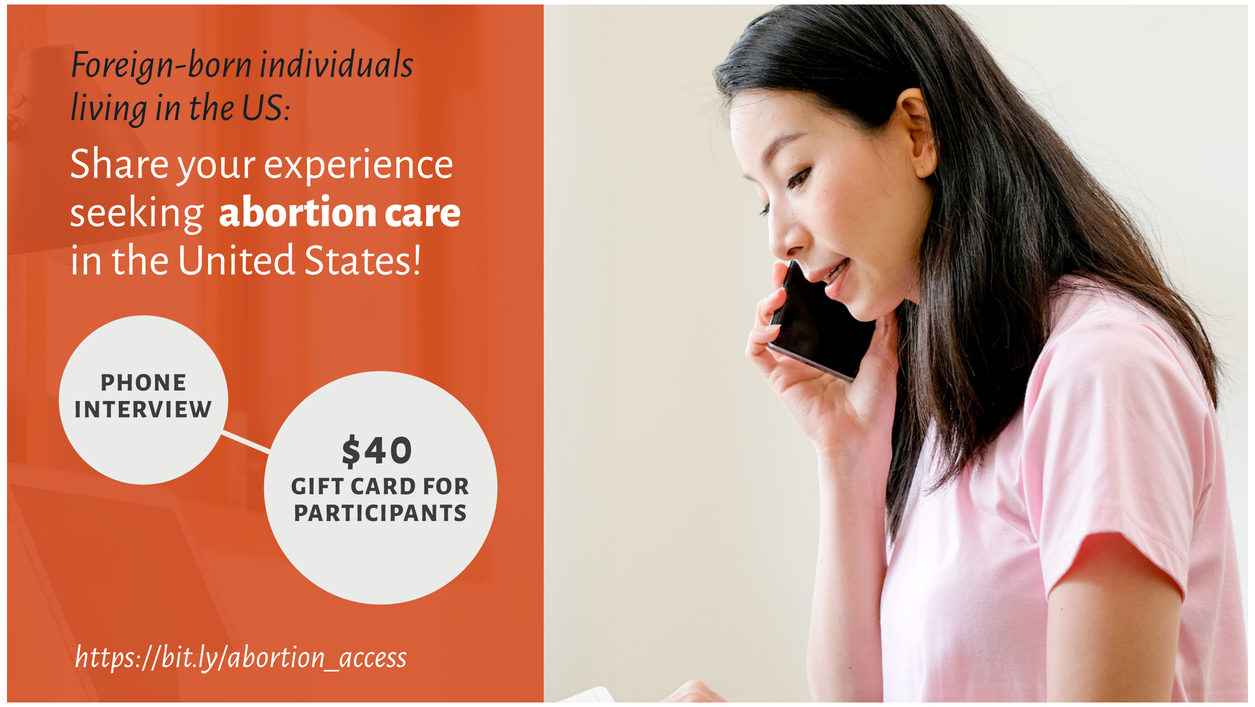
Foreign-born individuals living in the US:
Share your experience seeking **abortion care** in the United States!
PHONE INTERVIEW
$40 GIFT CARD FOR PARTICIPANTS
https://bit.ly/abortion_access

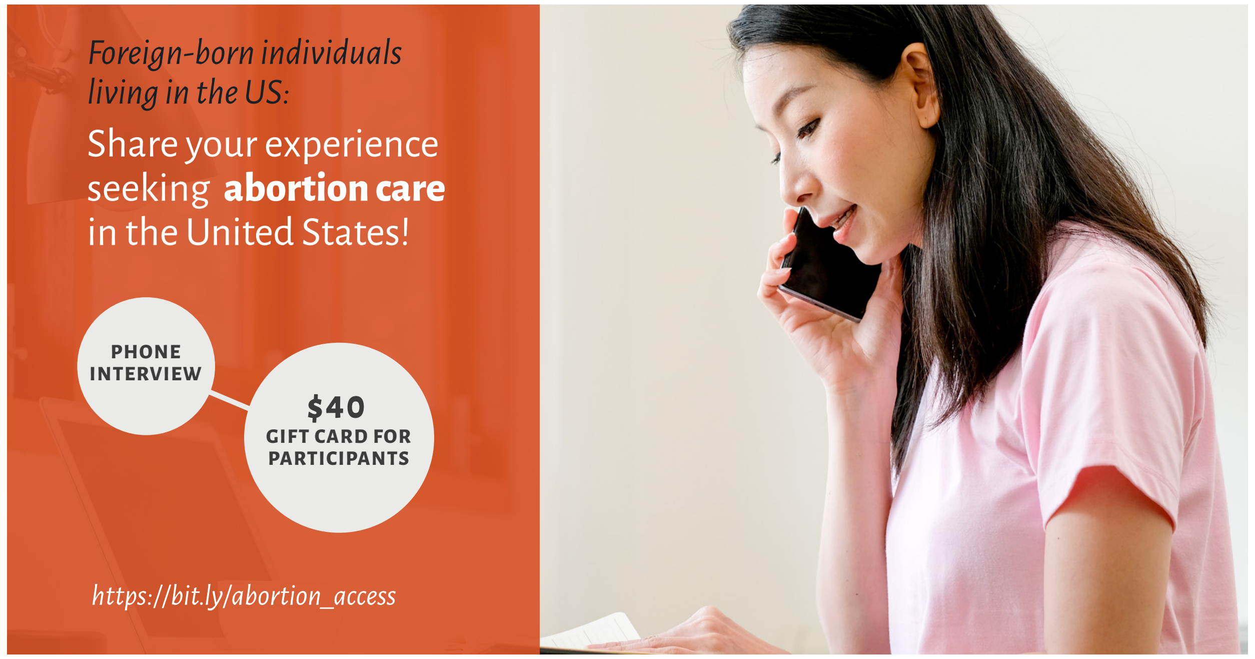
Foreign-born individuals living in the US:
Share your experience seeking **abortion care** in the United States!
PHONE INTERVIEW
$40 GIFT CARD FOR PARTICIPANTS
https://bit.ly/abortion_access

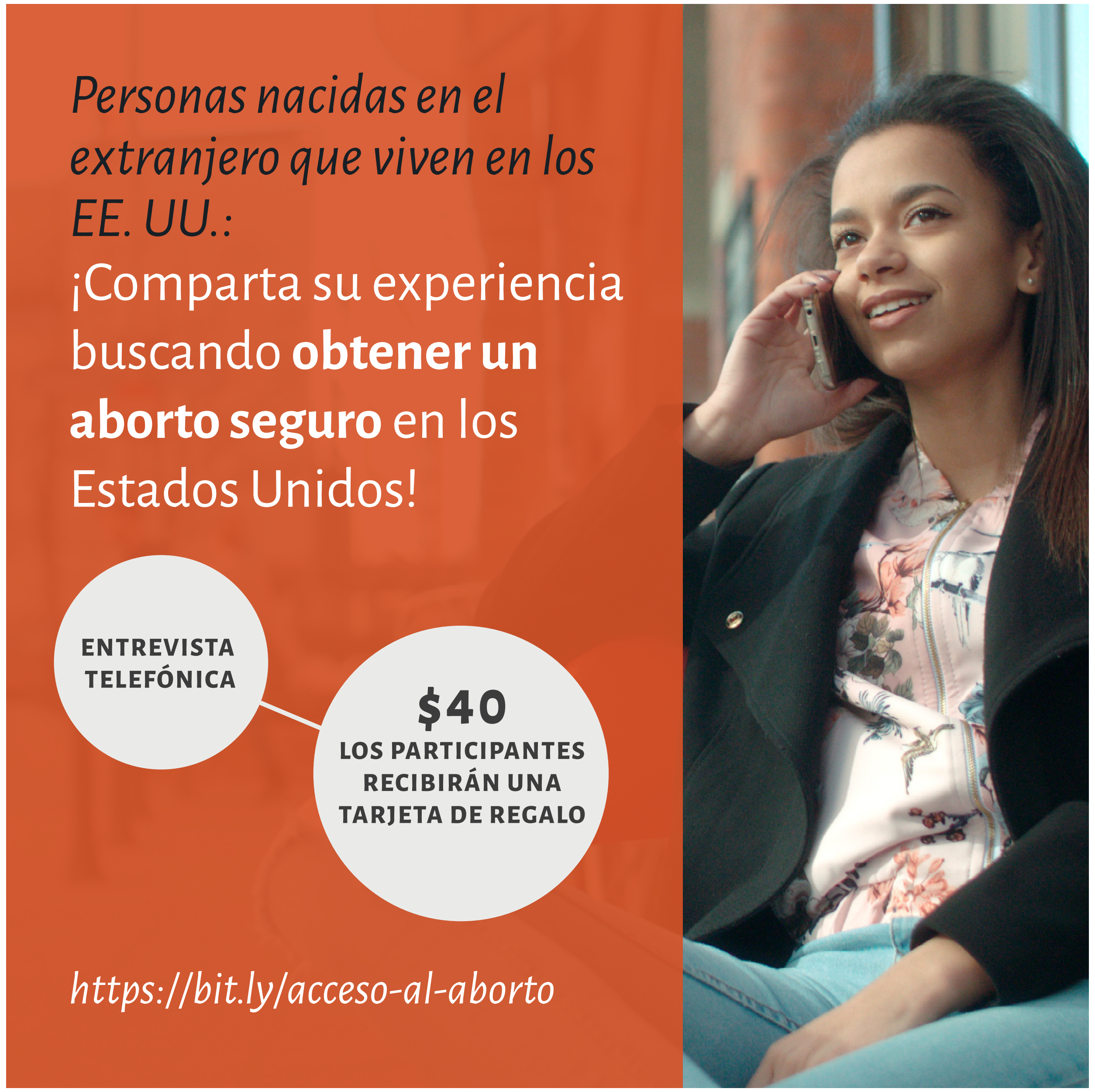
Personas nacidas en el extranjero que viven en los EE. UU.:
¡Comparta su experiencia buscando obtener un aborto seguro en los Estados Unidos!
ENTREVISTA TELEFÓNICA
$40
LOS PARTICIPANTES RECIBIRÁN UNA TARJETA DE REGALO
https://bit.ly/acceso-al-aborto

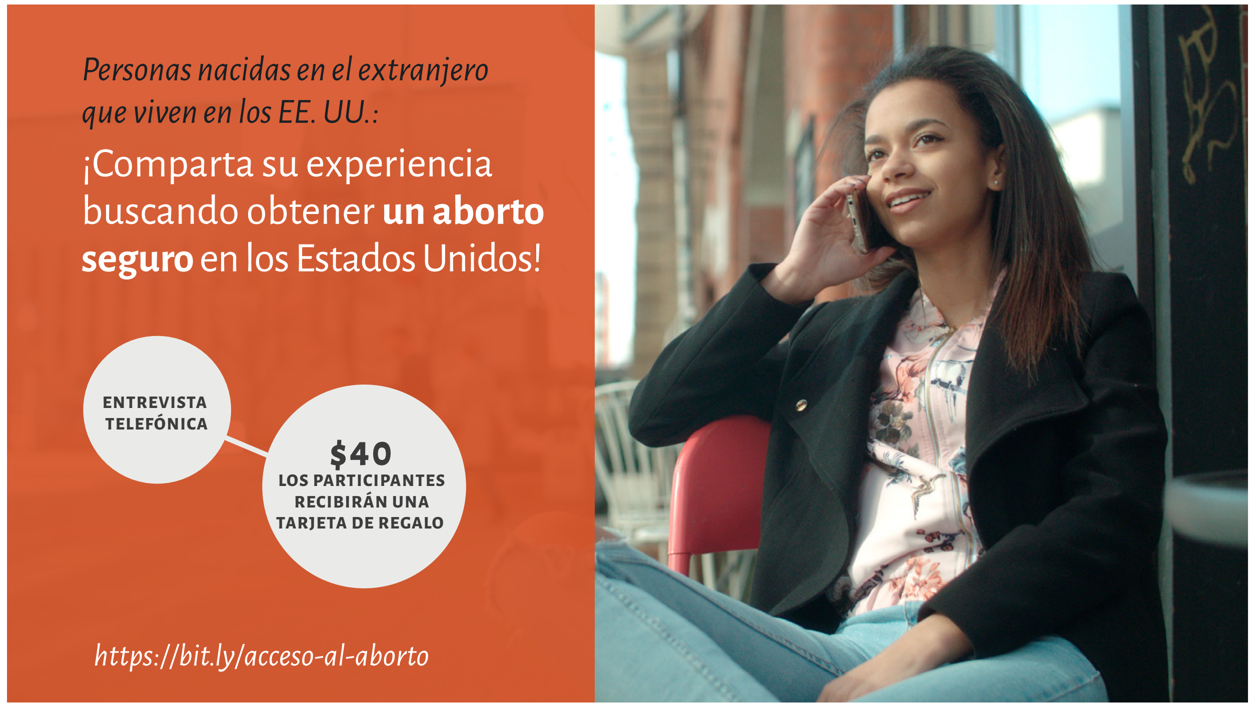
Personas nacidas en el extranjero que viven en los EE. UU.:
¡Comparta su experiencia buscando obtener un aborto seguro en los Estados Unidos!
ENTREVISTA TELEFÓNICA
$40
LOS PARTICIPANTES RECIBIRÁN UNA TARJETA DE REGALO
https://bit.ly/acceso-al-aborto

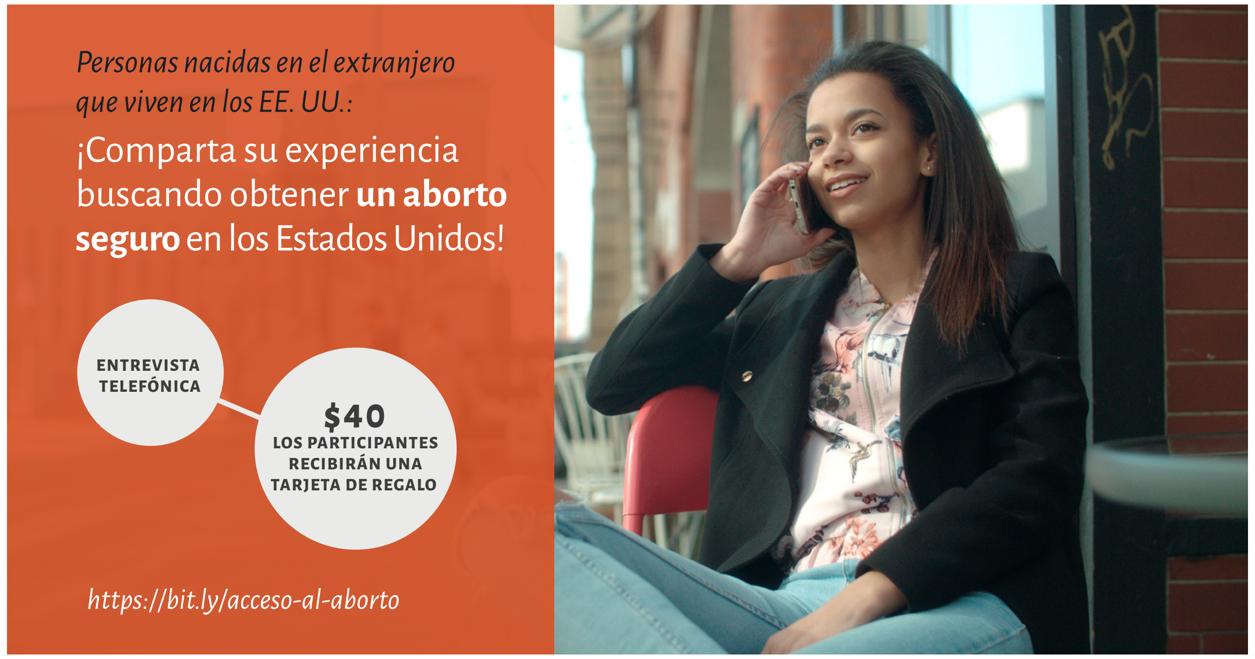
Personas nacidas en el extranjero que viven en los EE. UU.:
¡Comparta su experiencia buscando obtener un aborto seguro en los Estados Unidos!
ENTREVISTA TELEFÓNICA
$40
LOS PARTICIPANTES RECIBIRÁN UNA TARJETA DE REGALO
https://bit.ly/acceso-al-aborto

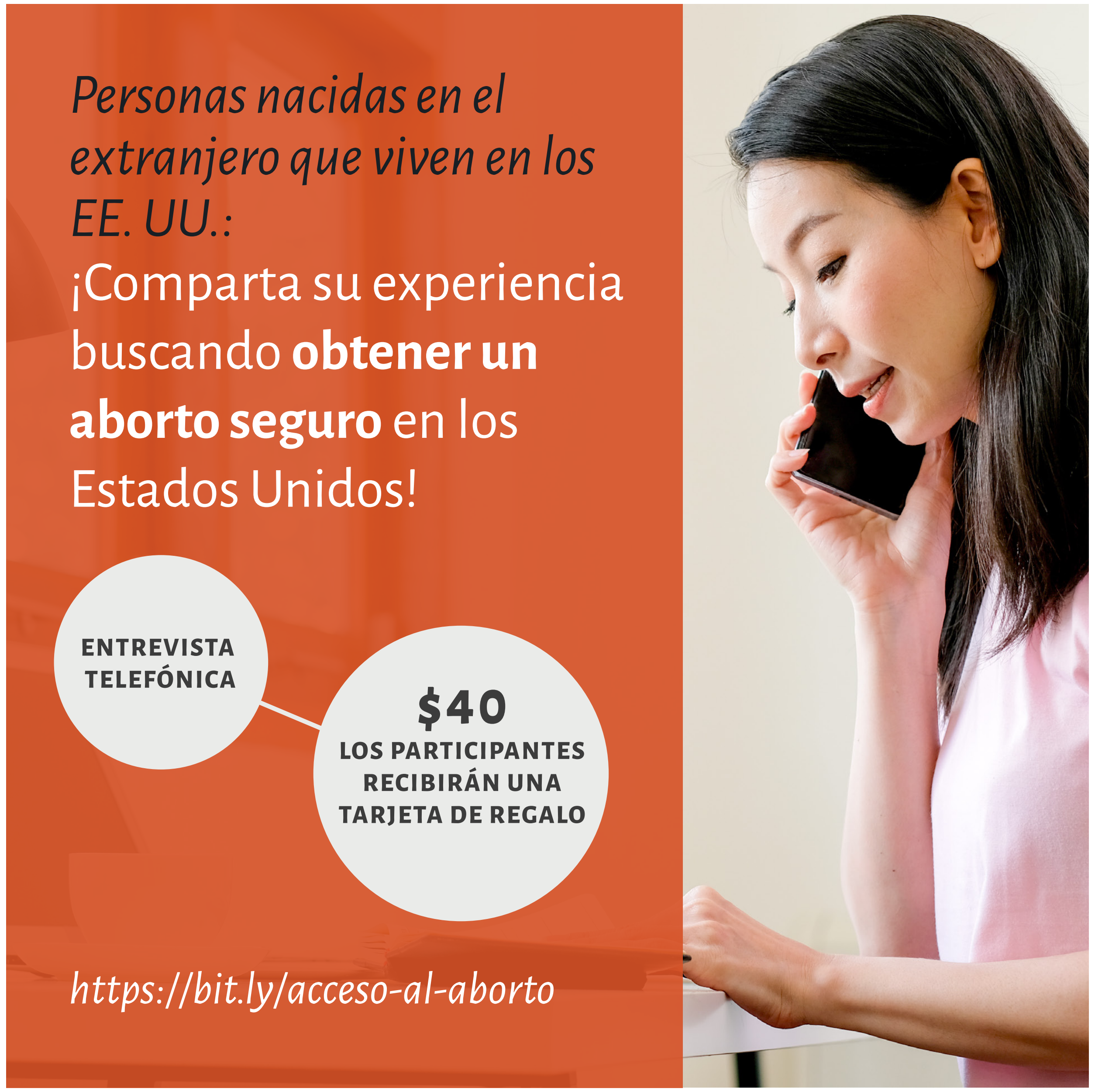
Personas nacidas en el extranjero que viven en los EE. UU.:
¡Comparta su experiencia buscando obtener un aborto seguro en los Estados Unidos!
ENTREVISTA TELEFÓNICA
$40
LOS PARTICIPANTES RECIBIRÁN UNA TARJETA DE REGALO
https://bit.ly/acceso-al-aborto

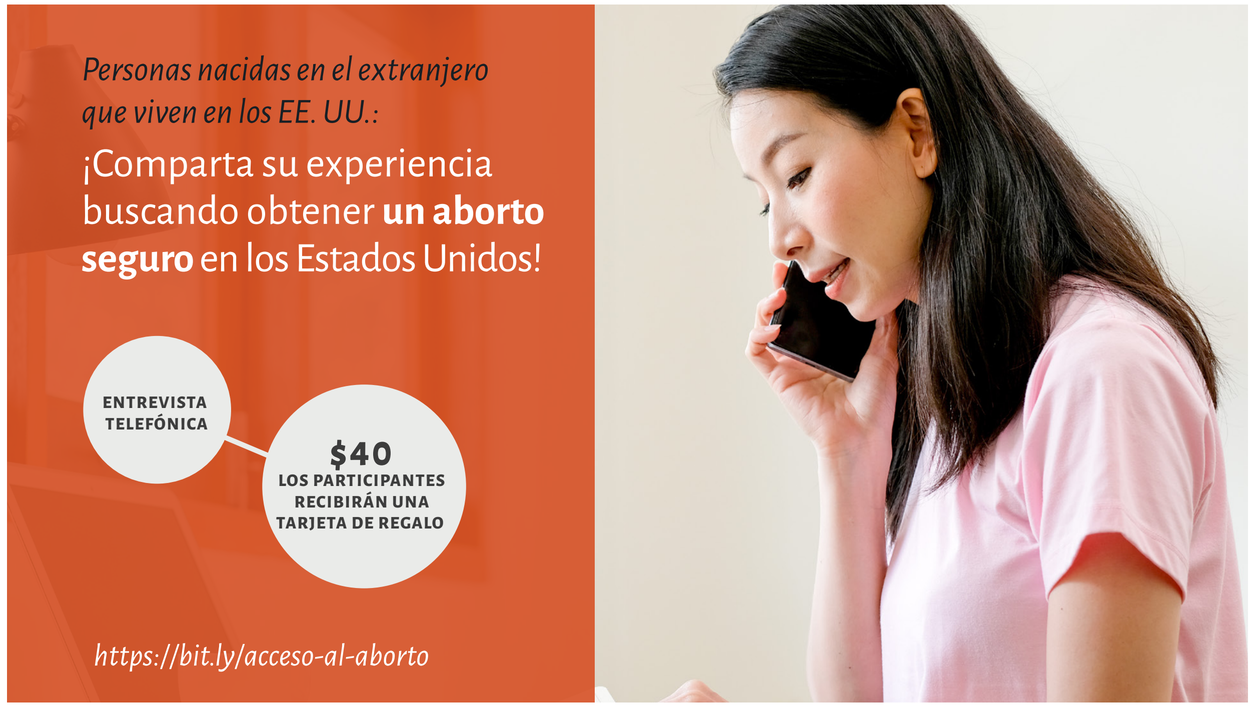
Personas nacidas en el extranjero que viven en los EE. UU.:
¡Comparta su experiencia buscando obtener un aborto seguro en los Estados Unidos!
ENTREVISTA TELEFÓNICA
$40
LOS PARTICIPANTES RECIBIRÁN UNA TARJETA DE REGALO
https://bit.ly/acceso-al-aborto

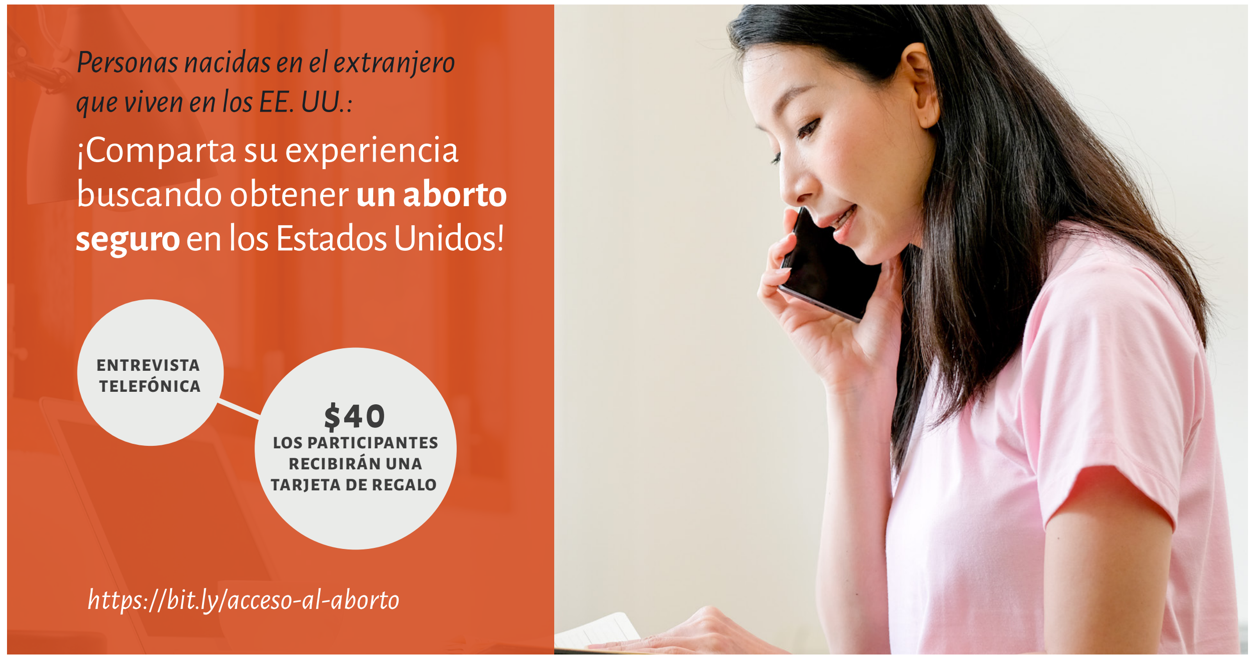
Personas nacidas en el extranjero que viven en los EE. UU.:
¡Comparta su experiencia buscando obtener un aborto seguro en los Estados Unidos!
ENTREVISTA TELEFÓNICA
$40
LOS PARTICIPANTES RECIBIRÁN UNA TARJETA DE REGALO
https://bit.ly/acceso-al-aborto
