# Supplementary material for: Recruiting foreign-born individuals who have sought an abortion in the United States: Lessons from a feasibility study
Source: Front Glob Womens Health. 2023 Apr 18;4:1114820. doi: 10.3389/fgwh.2023.1114820 (PMC10151930; doi:10.3389/fgwh.2023.1114820)
Supplement: Supplementary file 1 [file Datasheet1.zip › Appendix 2.DOCX]

**Appendix 2 – Study Webpage Text**

**A Study to Understand the Experiences of Foreign-born Abortion Seekers**

**What is the study about?**

People seeking abortion care in the United States may run into multiple barriers that delay or prevent them from receiving care, and although we know that foreign-born women obtain abortions at the same frequency as US-born women, we don’t know as much about the abortion seeking experiences of foreign-born individuals. In this study, we want to connect with a small, diverse group of foreign-born individuals who have sought an abortion in the United States in the past 2 years to understand their experiences. In particular, we are interested in exploring the issues/factors (good or bad) that foreign-born individuals encountered when attempting to access abortion services, and how these factors/issues could differ by age, race, country of origin, and length of time in the US. We also want to gather information about the experience of foreign-born individuals who have sought or obtained contraceptives services.

**Why participate?**

We don’t know much about the abortion seeking experiences of foreign-born individuals, so people who are willing to share their stories will help us understand any issues or factors (good or bad) they encountered when trying to access abortion services. Comparing the experiences of people of different backgrounds will also help us see if these factors/issues differ by age, race, country of origin, and length of time in the US. This information could help policies target and address barriers foreign-born individuals face to increase access to abortion. For sharing their stories with us, individuals who participate in the study will receive a $40 gift card of their choice for Amazon, Starbucks, or Target.

**How is the study being conducted?**

This study will gather information on the abortion-seeking experiences of foreign-born individuals through phone interviews and through an online demographic survey. Interviews will last 1-1.5 hours and can be done in either English or Spanish. No personally identifying information will be collected during the interview. After the interview, individuals will receive a link to complete a short online demographic survey and at the end of the survey, they can choose which gift card they would like to receive.

**Who can participate in the study?**

Individuals must:

- Have been born outside of the United States
- Currently live in the United States
- Have sought an abortion in the United States within the past 2 years (regardless of whether you obtained an abortion or not)
- Speak English or Spanish

**I’m interested in participating! What are the next steps?**

If you’re interested in participating in a phone interview, please fill out this brief questionnaire [link underlined text]. If you are eligible, you will view a form that tells you about your rights as a research participant. You will be asked to electronically agree to participate in the study. You will then be asked to fill out a short demographic questionnaire and can select your gift card preference at the end of the questionnaire. The questionnaire should take about 5 minutes to fill out. If selected, we will email you to schedule a phone interview.

**Other questions?**

If you have any questions about the study, please contact Carmela Zuniga at czuniga@ibisreprodutcivehealth.org.

**Un estudio para comprender las experiencias de las personas nacidas en el extranjero que han buscado servicios de aborto seguro**

**¿Sobre que es el estudio?**

Las personas que buscan servicios de aborto seguro en los Estados Unidos pueden encontrarse con múltiples barreras que retrasan o les impiden en recibir atención, y aunque sabemos que las mujeres nacidas en el extranjero obtienen abortos con la misma frecuencia que las mujeres nacidas en los Estados Unidos, no sabemos tanto sobre las experiencias de búsqueda de servicios de aborto seguro de personas nacidas en el extranjero. En este estudio, queremos conectarnos con un grupo pequeño y diverso de personas nacidas en el extranjero que han buscado servicios de aborto seguro en los Estados Unidos en los últimos 2 años para comprender sus experiencias. En particular, nos interesa explorar los problemas / factores (buenos o malos) que las personas nacidas en el extranjero encontraron al intentar acceder a los servicios de aborto seguro, y cómo estos factores / problemas podrían diferir según la edad, la raza, el país de origen y el tiempo que llevan en los Estados Unidos. También queremos recopilar información sobre las experiencias de personas nacidas en el extranjero que han buscado u obtenido servicios de anticonceptivos.

**¿Por qué participar?**

No sabemos mucho acerca de las experiencias de búsqueda de servicios de aborto seguro de personas nacidas en el extranjero, por lo que las personas que están dispuestas a compartir sus historias nos ayudarán a comprender cualquier problema o factor (bueno o malo) que encontraron al intentar acceder a los servicios de aborto seguro. Comparar las experiencias de personas de diferentes orígenes también nos ayudará a ver si estos factores / problemas difieren según la edad, la raza, el país de origen y el período de tiempo que llevan en los EE. UU. Esta información podría ayudar a las políticas a identificar y abordar las barreras que enfrentan las personas nacidas en el extranjero para aumentar el acceso al aborto. Por compartir sus historias con nosotrxs, las personas que participan en el estudio recibirán una tarjeta de regalo de $ 40 de su elección para Amazon, Starbucks o Target.

**¿Cómo se realiza el estudio?**

Este estudio reunirá información sobre las experiencias de búsqueda de aborto de personas nacidas en el extranjero a través de entrevistas telefónicas y una encuesta demográfica en línea. Las entrevistas durarán entre 1 hora y 1 hora y media y se pueden hacer en inglés o español. No se recopilará información de identificación personal durante la entrevista. Después de la entrevista, las personas recibirán un enlace para completar una breve encuesta demográfica en línea y, al final de la encuesta, pueden elegir qué tarjeta de regalo desean recibir.

**¿Quién puede participar en el estudio?**

Para ser elegible usted debe:

• Haber nacido fuera de los Estados Unidos.

• Vivir actualmente en los Estados Unidos

• Haber buscado servicios de aborto seguro en los Estados Unidos en los últimos 2 años (independientemente de si obtuvo un aborto o no)

• Hablar inglés o español

**¡Estoy interesada/o/e en participar! ¿Cuáles son los siguientes pasos?**

Si está interesada/o/e en participar en una entrevista telefónica, complete este breve cuestionario [enlace de texto subrayado]. Si es elegible, verá un formulario que le informa sobre sus derechos como participante de la investigación. Se le pedirá que acepte electrónicamente participar en el estudio. Luego se le pedirá que complete un breve cuestionario demográfico y puede seleccionar su preferencia de tarjeta de regalo al final del cuestionario. El cuestionario debe tomar alrededor de 5 minutos para completar. Si se selecciona, le enviaremos un correo electrónico para programar una entrevista telefónica.

**¿Otras preguntas?**

Si tiene alguna pregunta sobre el estudio, comuníquese con Sachiko Ragosta mandando un correo a sragosta@ibisreprodutcivehealth.org.
