# Supplementary material for: Recruiting foreign-born individuals who have sought an abortion in the United States: Lessons from a feasibility study
Source: Front Glob Womens Health. 2023 Apr 18;4:1114820. doi: 10.3389/fgwh.2023.1114820 (PMC10151930; doi:10.3389/fgwh.2023.1114820)
Supplement: Supplementary file 1 [file Datasheet1.zip › Appendix 3.DOCX]

**Appendix 3 – Screening Questionnaire**

**Thank you for your interest in participating in our study. Please answer the following questions so we can determine whether you can participate. We are trying to interview individuals with different backgrounds, which is why we are asking you questions about your age, race, country of origin, and length of time in the US. Your responses are confidential.**

**Screening Questions**

1. Have you ever tried to obtain an abortion in the United States?

- - Yes, in the past 2 years
  - Yes, over 2 years ago [ ineligible]
  - Yes, I’m currently trying get an abortion [ineligible]
  - No [Ineligible]

2. Were you able to obtain a wanted abortion in the United States?

- - Yes
  - No
  - I miscarried before I could get an abortion. [ineligible]

2. What country were you born in? [drop down list of countries]

- If US is selected ->[ineligible]

4. How long have you lived in the US? [text box]

5. How old are you? [Drop down 12-49]

6. Do you prefer to be interviewed in English or Spanish?

- English
- Spanish

7. How did you find out about this study? Select all that apply.

- A friend or family member told me about it
- Facebook
- Twitter
- Reddit
- On a webpage
  - Please specify which organization’s webpage:
- Other
  - Please specify:

[If eligible and 18 years or older] You are eligible to participate in our study! In order to participate, you will need to read the online form on the next page and agree to participate. After you have agreed to be in the study, we will ask you to provide some demographic information to help us understand how your experiences compare to other people of various backgrounds.

[If eligible and younger than 18 years old] You are eligible to participate in our study! In order to participate, you will need to read the online form on the next page and agree to participate. After you have agreed to be in the study, we will ask you to provide some demographic information to help us understand how your experiences compare to other people of various backgrounds.

[If not eligible] Unfortunately, you are not eligible to participate in this study. Thank you for your time and interest.

**Gracias por su interés en participar en nuestro estudio. Responda a las siguientes preguntas para que podamos determinar si es elegible para participar. Estamos tratando de entrevistar a personas con diferentes antecedentes, por eso le hacemos preguntas sobre su edad, raza, país de origen y tiempo en los Estados Unidos. Sus respuestas son confidenciales.**

**Preguntas para determinar elegibilidad:**

1. ¿Alguna vez ha intentado obtener un aborto en los Estados Unidos?

- Sí, en los últimos 2 años.
- Sí, hace más de 2 años [ no elegible]
- Sí, actualmente estoy intentando abortar [no elegible]
- No [ No elegible]

2. ¿Pudo obtener un aborto deseado en los Estados Unidos?

- Sí
- No
- Aborté antes de que pudiera abortar. [ no elegible]

3. ¿En qué país nació? [lista desplegable de países]

- Si se selecciona EE. UU. -> [no elegible]

4. ¿Cuánto tiempo ha vivido en los Estados Unidos? [caja de texto]

5. ¿Cuántos años tiene? [Desplegar 12-49]

6. ¿Prefiere ser entrevistada/o/e en inglés o español?

- inglés
- español

7. ¿Cómo se enteró de este estudio? [Seleccione todas las que correspondan]

- Un amigo o familiar me lo contó
- Facebook
- Twitter
- Reddit
- En una página web
  - Especifique la página web de la organización:
- Otro
  - Por favor especifique:

[Si es elegible y tiene 18 años o más] ¡Usted es elegible para participar en nuestro estudio! Para participar, deberá leer el formulario en línea en la página siguiente y aceptar a los términos de participación. Después de que haya aceptado participar en el estudio, le pediremos que brinde información demográfica para ayudarnos a comprender cómo se comparan sus experiencias con otras personas de diversos orígenes.

[Si es elegible y menor de 18 años] ¡Usted es elegible para participar en nuestro estudio! Para participar, deberá leer el formulario en línea en la página siguiente y aceptar a los términos de participación. Después de que haya aceptado participar en el estudio, le pediremos que brinde información demográfica para ayudarnos a comprender cómo se comparan sus experiencias con otras personas de diversos orígenes.

[Si no es elegible] Desafortunadamente, no es elegible para participar en este estudio. Gracias por su tiempo e interés.
