# Supplementary material for: Recruiting foreign-born individuals who have sought an abortion in the United States: Lessons from a feasibility study
Source: Front Glob Womens Health. 2023 Apr 18;4:1114820. doi: 10.3389/fgwh.2023.1114820 (PMC10151930; doi:10.3389/fgwh.2023.1114820)
Supplement: Supplementary file 1 [file Datasheet1.zip › Appendix 4.DOCX]

**Appendix 4 - In-Depth Interview Guide**

**Background Info**

**I’m going to start the interview by asking a few questions about your abortion experience.**

1. How many times have you wanted an abortion while living in the United States?

- Of the X times you wanted an abortion, were you able to get one?
- What happened the other Y times? (*probe to see if they attempted to access abortion in these circumstances)*

2. [If yes, they had an abortion] Were you able to get the abortion(s) you wanted in the United States?

- [If no] Did you get the abortion[s] outside the US?

**Thank you for sharing that information. I want you to focus on the last time you sought an abortion. Now let’s start at the beginning, when you found out about the pregnancy.**

- Do you remember which year and month that was? *(probe if they attempted to get an abortion during COVID-19 pandemic)*

**Can you tell me more about your decision-making process? How did you come to decide that you wanted an abortion?**

*Probes*

- Did your personal views on abortion make the decision process particularly easy or difficult?
- What did you know about abortion at the time you were considering it as an option?
  - Did you look up any information before making a decision?
    - If so, what kind of information did you search for?
    - If so, how did this impact your decision?
- Were there any other factors or life circumstances that made the decision process particularly easy or difficult?
  - [If during pandemic]: Did the pandemic influence your decision-making process in any way?
- Did anyone talk to you about abortion? If so – what did they talk to you about?
  - Did you get a sense that they had opinions (moral or emotional or otherwise) about abortion as an option?
  - Did their opinion influence the way you thought about abortion as an option?
- Were you using birth control at the time you got pregnant?
- How many weeks were you pregnant when you decided you wanted an abortion?

4. Once you knew you wanted an abortion, what did you do? Can you walk me through the steps you took and who was involved?

*Access probes*

- Did you know where you could get an abortion?
  - How near or far was the nearest place? What did this mean for you in terms of transportation, time off work, child care, etc.?

[If during pandemic]: Was the [transportation/time off work/child care] needed to get to an abortion impacted by the pandemic or pandemic related policies? If so, how?

*Knowledge probes*

- Did you know of any state laws that would impact your ability to have an abortion?
- Did you know about the different abortion procedures? If so, did you know what kind of abortion you wanted/needed?
- Did you ever encounter a language barrier when trying to obtain information about an abortion?
- How did your knowledge about abortion impact your ability to access abortion care?
- [If during pandemic]: Did you know of any pandemic-related policies that would impact your ability to have abortion?
  - How easy or difficult was it to find information about how pandemic-related polices impacted clinics and abortion care?

*Financial probes*

- Did you know how much the abortion you wanted/needed cost?
- Did you have insurance at the time?
  - [If insurance] Did you know or try to find out if your insurance covered abortions?
- Did the cost of the abortion impact your ability to access abortion care?
  - How easy or difficult was it to come up with the money to pay for the procedure?
- If covid: Did the pandemic or pandemic-related policies impact your ability to pay for an abortion?

*Other People*

- Did friends or family members or anyone else know you were trying to get an abortion?
- Did this person/these people try to help or support you getting an abortion?
- Did this person/these people try to prevent you from getting an abortion?
- [If during pandemic]: Did social distancing measures due to the pandemic influence who you spoke with about trying to get an abortion?

[If no abortion]

- What state were you living in when you tried to get an abortion?
- To summarize, what would you say was the main reason you did not get an abortion for that pregnancy?
- Were you able to access prenatal care afterwards?
  - - If no, why weren’t you able to access prenatal care?
    - If yes, was it an easy or difficult process to access prenatal care?
    - [If during pandemic]: Did the pandemic or pandemic-related policies impact your ability to access care? If so, how?

[If participant had an abortion]

- Where did you get your abortion? Probe for: clinic, hospital, self-induced?
  - [If during pandemic]: Was this location influenced by the pandemic?

[If self-induced]

- Can you describe how you carried out the abortion?

*If pills:*

- - Where did you get the pills from?
  - Were they easy to obtain?
  - How much did they cost? Could you use insurance?
  - How were you instructed to take the pills?
  - How did you feel about taking them?
  - Was anyone there with you during the time you took the pills?
  - [If during pandemic]: Did the pandemic or pandemic-related policies impact the way you carried out the abortion?

*If participant attempted an unsafe abortion/did not use pills*

- Can you describe how you carried out the abortion?
- How did you find out about this method?
- What happened after this attempt?
- [If during pandemic]: Did the pandemic or pandemic-related policies impact the way you carried out the abortion?

[If clinic or hospital]

- In which state did you have the abortion? Was this the same state where you were living?
- How many weeks pregnant were you when you made the appointment?
  - [If during pandemic]: Did the pandemic or pandemic-related polices impact when you made the appointment?
- How many weeks pregnant were you when you got the abortion?
  - [If during pandemic]: Was this impacted by the pandemic?
- How easy or difficult was it to make an appointment?
  - [If during pandemic]: Was this impacted by the pandemic?
- How did you feel about making an appointment?
- Was it easy to get to the clinic/hospital?
  - [If during pandemic]: Was this impacted by the pandemic?
- How did you feel when you were at the clinic for your abortion appointment?
  - Did you feel like you were being treated differently from other patients for any reason?
- How did you pay for the abortion?
  - [If during pandemic]: Was this impacted by the pandemic?
- How did you feel after getting the abortion?
- Were you offered any contraceptive counseling after the abortion?
- Did you want information about birth control after your abortion?

*Other Barriers or Facilitators*

- Were there any other factors that made it difficult to get an abortion that we haven’t already discussed?
- [If abortion] Were there any other factors that made it easy to get an abortion that we haven’t already discussed?

[If participant mentioned wanting more than 1 abortion] You mentioned you wanted more than 1 abortion while living in the US. Were there any different barriers or facilitators that impacted your ability to get an abortion for those pregnancies?

**Post Abortion**

- Was there any information or resources you wished you had that would have made your abortion seeking experience better?
- Is there any information or advice you would give to other foreign-born people seeking abortion?

**Networks**

Have you sought support or information, either in-person or online, from others who have had an abortion or tried to get an abortion?

**Contraceptive Questions**

While in the US, have you used a birth control method to prevent pregnancy?

*If no*

- Can you tell me the reasons for why you chose not to use birth control to prevent pregnancy in the US?

*If yes*

- What types of contraceptives have you used?
- Were any of these methods more difficult or easy to obtain than other methods? What made them difficult/easy to obtain?
- [If multiple methods/switched methods] What made you switch to a different method?
- Are they any methods you are interested in using, but haven’t been able to?
  - Can you describe why you haven’t been able to try this/these methods?
- Are you using any type of contraceptive method now?
  - How did you come to use your current method of contraception?
  - Are you happy with your current method?
- Where do you get information about contraceptives?

**Thank you so much for taking the time to speak with me! If you know of anyone else born outside of the US and who has tried to get an abortion in the US, please let them know about this study!**

**In-Depth Interview Guide**

**Background Info**

**Voy a comenzar la entrevista haciendo algunas preguntas sobre su experiencia relacionado con el aborto.**

1. ¿Cuántas veces ha buscado obtener un aborto seguro mientras vivía en los Estados Unidos?

- De las X veces que buscaba obtener un aborto seguro, ¿pudo conseguirlo?
- ¿Qué pasó las otras veces? (investigue si intentaron acceder al aborto en estas circunstancias)

2. [*En caso afirmativo, que tuvo un aborto*] ¿Pudo obtener el (los) aborto (s) que quería en los Estados Unidos?

- [*Si no*] ¿Recibió el / los aborto (s) fuera de los Estados Unidos?

**Gracias por compartir esta información. Quiero que se concentre en la última vez que buscó obtener un aborto. Ahora comencemos por charlar sobre la situación en que estaba cuando se enteró del embarazo.**

- ¿Recuerda en qué año y mes ocurrió? (investigue si intentaron abortar durante la pandemia de COVID-19)

**¿Me puede decir más sobre su proceso para la toma de decisiones? ¿Cómo llegó a decidir que quería obtener un aborto seguro?**

*Probes*

- ¿Sus puntos de vista personales sobre el aborto hicieron que el proceso de decisión fuera particularmente fácil o difícil?
- ¿Qué sabía sobre el aborto en el momento en que lo estaba considerando como una opción?
  - ¿Buscó alguna información antes de tomar una decisión?
    - Si es así, ¿qué tipo de información buscó?
    - Si es así, ¿cómo impactó esto en su decisión?
- ¿Hubo otros factores o circunstancias de la vida que complicaron o facilitaron el proceso de decisión?
  - [Si durante la pandemia]: ¿Influyó la pandemia su proceso de toma de decisiones de alguna manera?
- ¿Alguien le habló sobre el aborto? Si es así, ¿de qué le hablaron?
  - ¿Tuvo la sensación de que tenían opiniones (morales, emocionales o de otro tipo) sobre el aborto como opción?
  - ¿Su opinión influyó la forma en que pensaba usted sobre el aborto como una opción?
- ¿Estaba usando anticonceptivos cuando se quedó embarazada/e/o?
- ¿Cuántas semanas estaba embarazada/e/o cuando decidió que quería obtener un aborto seguro?

4. Una vez que supo que quería un aborto, ¿qué hizo? ¿Puede guiarme por los pasos que tomó y quién estuvo involucrada/e/o?

*Access probes*

- ¿Sabía dónde podía abortar de forma segura?
  - - - ¿Qué tan lejos estaba el lugar más cercano? ¿Qué significó esto para usted en términos de transporte, tiempo libre, cuidado de niños, etc.?

[*Si durante la pandemia*]: ¿Fue impactada/e/o por la pandemia o las políticas relacionadas con la pandemia, el [transporte / tiempo libre / cuidado infantil] necesario para poder acceder a los servicios de aborto seguro? ¿Si es así, cómo?

*Knowledge probes*

- ¿Sabía usted de alguna ley estatal que afectaría su habilidad de abortar?
- ¿Sabía sobre las diferentes opciones para realizar un aborto? Si es así, ¿sabía qué tipo de aborto deseaba / necesitaba?
- ¿Alguna vez encontró una barrera con el idioma al intentar obtener información sobre el aborto?
- ¿Cómo su conocimiento sobre el aborto impactó su habilidad de acceder a la atención del aborto?
- [Si durante la pandemia]: ¿Sabía de alguna política relacionada con la pandemia que afectaría su habilidad de acceder al aborto seguro?
  - - - ¿Qué tan fácil o difícil fue encontrar información sobre cómo las políticas relacionadas con la pandemia afectaron las clínicas y los servicios de aborto seguro?

*Financial probes*

- ¿Sabía el costo del procedimiento de aborto que deseaba / necesitaba?
- ¿Tenía seguro en ese momento?
  - - - [Si tenía seguro] ¿Sabía o intentó averiguar si su seguro cubría el aborto?
- ¿El costo del aborto afectó su posibilidad para acceder a la atención del aborto?
  - - - ¿Qué tan fácil o difícil fue obtener el dinero para pagar el procedimiento?
- *If during covid*: ¿La pandemia o las políticas relacionadas con la pandemia afectaron su capacidad de pagar por un aborto?

*Other people*

- ¿Sabían sus amigos/as/es, familiares o alguien más que estaba intentando abortar?
  - - - ¿Esta persona / estas personas trataron de ayudarle o apoyarle para acceder a un aborto seguro?
      - ¿Esta persona / estas personas intentaron evitar que abortara?
      - [Si durante la pandemia]: ¿Las medidas de distanciamiento social debido a la pandemia influyeron con quién habló sobre el intento de abortar?

*[Si no obtuvo un aborto*]

- ¿En qué estado vivía cuando intentó abortar?
- En resumen, ¿cuál diría que fue la razón principal por la que no accedió a un aborto en esa ocasión?
- ¿Pudo acceder a la atención prenatal después?
  - Si no, ¿por qué no pudo acceder a la atención prenatal?
  - En caso que sí pudo, ¿fue un proceso fácil o difícil acceder a la atención prenatal?
  - [Si durante la pandemia]: ¿La pandemia o las políticas relacionadas con la pandemia afectaron su posibilidad de acceder a la atención? ¿Si es así, cómo?

*[Si la participante tuvo un aborto]*

- ¿Dónde accedió a servicios de aborto seguro? *Probe for*: clínica, hospital, autoinducido?
  - [Si durante una pandemia]: ¿Fue esta localidad influenciada por la pandemia?

*[Si fue autoinducido]*

- ¿Puedes describir cómo se llevó a cabo el aborto?

*Si pastillas:*

- - - - - ¿Dónde consiguió las pastillas?
        - ¿Fue fácil obtenerlas?
        - ¿Cuánto le costaron? ¿Pudo usar un seguro de salud?
        - ¿Cuales fueron las indicaciones para tomar las pastillas?
        - ¿Cómo se sintió al tomarlas?
        - ¿Hubo alguien con usted durante el tiempo que tomó las pastillas?
        - [Si durante la pandemia]: ¿La pandemia o las políticas relacionadas con la pandemia afectaron la forma en que llevó a cabo el aborto?

*Si la participante intentó un aborto inseguro / no usó pastillas*

- ¿Puede describir cómo se llevó a cabo el aborto?
- ¿Cómo se enteró de este método?
- ¿Qué pasó después? ¿Cómo resultó?
- [Si durante la pandemia]: ¿La pandemia o las políticas relacionadas con la pandemia afectaron la forma en que se llevó a cabo el aborto?

*[If clinic or hospital]*

- ¿En qué estado tuvo el aborto? ¿Era este el mismo estado donde vivías?
- ¿Cuántas semanas de embarazo tenía cuando hizo la primera cita?
  - [Si durante la pandemia]: ¿La pandemia o las políticas relacionadas con la pandemia impactaron cuando hizo la cita?
- ¿Cuántas semanas de embarazo tenía cuando abortó?
  - [Si durante la pandemia]: ¿Se vio afectada/e/o por la pandemia?
- ¿Qué tan fácil o difícil fue hacer una cita?
  - [Si durante la pandemia]: ¿Se vio afectada/e/o por la pandemia?
- ¿Cómo se sintió al hacer una cita?
- ¿Fue fácil llegar a la clínica / hospital?
  - [Si durante la pandemia]: ¿Se vio afectada/e/o por la pandemia?
- ¿Cómo se sintió cuando estaba en la clínica para su primera cita de aborto?
  - ¿Sintió que estaba siendo tratada/e/o de manera diferente a otrxs pacientes por alguna razón?
- ¿Cómo pagó el aborto?
  - [Si durante la pandemia]: ¿Se vio afectada/e/o por la pandemia?
- ¿Cómo se sintió después de haber realizado el aborto?
- ¿Le ofrecieron asesoramiento anticonceptivo después del aborto?
- ¿Quería información sobre anticonceptivos después de su aborto?

*Other Barriers or Facilitators*

- ¿Hubo otros factores que dificultaron la realización de un aborto que aún no hemos discutido?
- [Si el aborto] ¿Hubo otros factores que facilitaron el aborto que aún no hemos discutido?

[*If participant mentioned wanting more than 1 abortion]* Usted mencionó que quería más de 1 aborto mientras vivía en los Estados Unidos. ¿Hubo barreras o facilitadores que no ha mencionado que afectaron su posibilidad de acceder a un aborto seguro en cuanto a esos embarazos?

**Post Abortion**

- ¿Hubo alguna información o recursos que le gustaría haber tenido para mejorar su experiencia de búsqueda de acceso al aborto?
- • ¿Hay alguna información o consejo que le daría a otras personas nacidas en el extranjero que buscan acceder a un aborto seguro?

**Networks**

¿Ha buscado apoyo o información, ya sea en persona o en línea, de otras personas que han podido o intentado acceder a un aborto?

**Contraceptive Questions**

Durante su tiempo viviendo en los Estados Unidos, ¿ha utilizado un método anticonceptivo para prevenir el embarazo?

*If no*

- ¿Puede contarme sobre las razones por las que eligió no usar un método anticonceptivo para prevenir el embarazo en los Estados Unidos?

*If yes*

- ¿Qué tipos de anticonceptivos ha usado?
- ¿Alguno de estos métodos fue más difícil o fácil de obtener que otros métodos? ¿Qué los hizo difíciles / fáciles de obtener?
- [*Si hay varios métodos / ha cambiado de método*] ¿Qué le hizo cambiar a un método diferente?
- ¿Hay algún método que le interese usar, pero no ha podido?
  - ¿Puede describir por qué no ha podido probar este / estos métodos?
- ¿Está utilizando algún tipo de método anticonceptivo actualmente?
  - - - - ¿Cómo llegó a usar su método anticonceptivo actual?
        - ¿Está contenta/e/o con su método actual?
- ¿Dónde obtiene información sobre anticonceptivos?

**¡Muchas gracias por tomarse el tiempo de hablar conmigo! Si conoce a otra persona nacida fuera de los EE. UU. que haya intentado abortar en los EE. UU., ¡Infórmeles sobre este estudio si puede!**
