# Supplementary material for: Recruiting foreign-born individuals who have sought an abortion in the United States: Lessons from a feasibility study
Source: Front Glob Womens Health. 2023 Apr 18;4:1114820. doi: 10.3389/fgwh.2023.1114820 (PMC10151930; doi:10.3389/fgwh.2023.1114820)
Supplement: Supplementary file 1 [file Datasheet1.zip › Appendix 5.DOCX]

**Appendix 5 - Resource guide**

Abortion and Sexual and Reproductive Health Counseling

**For support around a current pregnancy, past pregnancy, or pregnancy-related experience**, call the [All-Options Talkline](https://www.all-options.org/find-support/talkline/) at 1-888-493-0092. Peer counselors can help you decide what the best decision is for you or provide emotional support for a past pregnancy experience.

If you know you want an abortion, you have a few options depending on gestational age and your preferences around privacy, clinic involvement, etc.:

- **Medication Abortion** (MA) is available in most abortion clinics for up to 7-10 weeks of pregnancy. MA involves taking pills orally and then passing the pregnancy at home. Side effects include bleeding and cramping that could last between a few hours to a few days.
- **Vacuum Aspiration** is usually used up to 14-16 weeks of pregnancy. During this procedure, a clinician opens the cervix and may use anesthesia to help with the pain before using a device to remove the pregnancy. This procedure is quick and can be completed in a single clinic visit.
- **Dilation and Evacuation** (D&E) is usually used for pregnancies over 14 weeks. During this procedure the clinician may use dilator sticks in a first visit before removing the pregnancy with medical instruments and/ or a suction device in a later visit.

If you are unsure **which method is right for you,** visit [Pregnancy Options online.](http://www.pregnancyoptions.info/whichmethod.htm)

**To find an abortion care provider** in your area, you can use one of the following clinic finder tools:

- Abortion Care Network Member [Clinic Finder](https://abortioncarenetwork.org/find-a-clinic/)
- National Abortion Federation [Find A Provider](https://prochoice.org/patients/find-a-provider/) or you can call 1-877-257-0012 if you have been diagnosed with a fetal abnormality or otherwise require specialized later abortion care
- [I Need an A](https://ineedana.com/)
- [Find a Planned Parenthood health center](https://www.plannedparenthood.org/health-center) or call 1-800-230-PLAN

If you need support with miscarriage or a self-managed abortion you can contact the [M+A Hotline](https://www.mahotline.org/) at 1-833-246-2632

Know your rights!

While abortion is legal across the United States, there are many state-by-state policies and restrictions in place that can limit access to an abortion. This [resource](https://www.guttmacher.org/state-policy/explore/overview-abortion-laws) includes a chart of the restrictions in each state. For additional legal support, you can call the following helplines:

- [If/When/How Repro Legal](https://www.reprolegalhelpline.org/) Helpline provides free information on abortion law and self-managed abortion. You can call at 1-844-868-2812 or you can fill out the [online form](https://www.reprolegalhelpline.org/contact-the-helpline/#secure-form) with your question(s).
- [Immigrant Defense Project](https://www.immigrantdefenseproject.org/what-we-do/legal-advice/) provides legal assistance to immigrants through the helpline at 1-212-725-6422

Funding support

Cost for the abortion as well as things like travel and accommodations, can be a huge barrier to abortion services, but there are many organizations out there dedicated to supporting abortion clients. The [National Network of Abortion Funds](https://abortionfunds.org/need-abortion/) can help connect you to **local financial and logistical support for your abortion**. On the website, enter your address to find an abortion fund near you.

For **financial assistance** you can also call the National Abortion Federation at 1-800-772-9100.

Mental Health services:

This [Comprehensive Mental Health Guide](https://www.informedimmigrant.com/resource-type/mental-health/) by Informed Immigrant includes directories of mental health providers, information on mental health disorders and coping toolkits.

There are a number of additional directories and resource guides including:

- [Black Mental Wellness](https://www.blackmentalwellness.com/), information and resources for Black individuals
- [Therapy for Latinx](https://www.therapyforlatinx.com/), a directory of Latinx mental health providers and additional resources
- [The National Asian American Pacific Islander Mental Health Association](https://www.naapimha.org/aanhpi-service-providers), resource list and localized programs
- [The South Asian Mental Health Initiative & Network](https://samhin.org/), provider network, helpline, resources and a support group

Other Resources

RAICES [Canopy Hotline](https://www.raicestexas.org/what-we-do/social-services/canopy-hotline/) **connects immigrants directly to immigrant friendly services** all across the United States including to reproductive health care. You can call or text: 1-800-437-3071

**Support for Lesbian, Gay, Bisexual, Transgender and Queer (LGBTQ+ ) Asylum seekers** during COVID-19 can be found in this [resource list](https://asylumconnect.org/resource-list-for-lgbtq-asylum-seekers-and-other-lgbtq-people-during-covid-19/) compiled by Asylum Connect.

**Download** [**Euki**](https://womenhelp.org/en/page/1082/euki-app), a reproductive and sexual health app, to track period symptoms, sexual health experiences and symptoms, take a quiz to find the right contraceptive method for you and learn about gender, sexuality, pregnancy, abortion, and more!

Boston- Local Resources

COVID-19 related resources for immigrants and refugees through the [Massachusetts Immigrant & Refugee Advocacy Coalition](https://www.miracoalition.org/resources/covid19/). This organization also provides resources more broadly for immigrants and refugees in Massachusetts including legal services, health services, educational and citizenship services.

[Fenway Health](https://fenwayhealth.org/) offers a diversity of physical and behavioral health care services inclusive of LGBTQ+ individuals in the greater Boston area, including primary care, free STI screening, transgender affirming services, contraceptive counseling, and therapy.

The [Immigrant and Refugee Health Center](https://www.bmc.org/immigrant-refugee-health-center) is a part of Boston Medical Center that provides comprehensive health and social services to immigrants.
