# Supplementary material for: Recruiting foreign-born individuals who have sought an abortion in the United States: Lessons from a feasibility study
Source: Front Glob Womens Health. 2023 Apr 18;4:1114820. doi: 10.3389/fgwh.2023.1114820 (PMC10151930; doi:10.3389/fgwh.2023.1114820)
Supplement: Supplementary file 1 [file Datasheet1.zip › Appendix 6.DOCX]

**Appendix 6 - Online Survey Questions**

**Background Info**

- How many times have you **tried to get an** abortion while living in the United States, even if you ended up not getting one? [Drop-down 1-10+]
- How many abortions have you had in the United States? [Drop down 0-5+]
- [If not 0] In which state(s) have you gotten an abortion? Please select all that apply. [Drop down states]

**For the following questions, please think about the most recent time you tried to get an abortion.**

- How many weeks pregnant were you when you found out you were pregnant? [drop-down 1-40]
- What state were you living in at this time? [drop down]
- Did you try to get an abortion after March 13, 2020 (since the start of the COVID-19 pandemic)?
  - Yes
  - No
- When you found out you were pregnant, did you know right away that you would try to get an abortion?
  - Yes
  - No
  - I don’t remember
- What did you first do when you found out you were pregnant?
  - Tried to get more information about abortion
  - Talked with or reached out to someone
  - Made an appointment
  - Took some time to think
  - Other (please tell us):
  - I don’t remember

[If online research]: Please tell us what you researched:

[If talked to someone AND didn’t know right away OR Unsure] Who did you first want to talk with when trying to decide if abortion was the right decision for you?

- Partner
- Family member
- Friend
- Provider
- Other

[If provider] Please tell us what type of provider you spoke with (for example, did you speak with an abortion provider? A family doctor? A pharmacist?) (open-ended)

[If talked to someone AND didn’t know right away OR unsure] Did you talk to anyone else while trying to decide to have an abortion?

- Yes -> please tell us:
- No
- Don’t remember

[If talked to someone AND didn’t know right away OR unsure AND during pandemic]: Did social distancing measures or recommendations due to the pandemic influence who you spoke with about trying to get an abortion?

- - Yes
  - No
  - Unsure

[If talked to someone and knew right away] Who did you first want to talk with about your decision to have an abortion?

- Partner
- Family member
- Friend
- Provider
- Other

[If provider] Please tell us what type of provider you spoke with (for example, did you speak with an abortion provider? A family doctor? A pharmacist?) (open-ended)

[If talked to someone AND knew right away] Did you talk to anyone else about your decision to have an abortion?

- Yes -> please tell us:
- No
- Don’t remember

[f talked to someone AND knew right away AND during pandemic]: Did social distancing measures or recommendations due to the pandemic influence who you spoke with about your decision to get an abortion?

- - Yes
  - No
  - Unsure

[If talked to someone] Did anyone’s opinion make you more sure about getting an abortion?

- Yes
- No
- Unsure
- Don’t remember

[If talked to someone] Did anyone’s opinion make you no longer want to get an abortion?

- Yes
- No
- Unsure
- Don’t remember
- How did your personal views on abortion impact your decision to try to get an abortion?
  - It made the decision very easy
  - It made the decision somewhat easy
  - It made the decision somewhat difficult
  - It made the decision very difficult
  - My personal views did not impact my decision at all
  - I’m unsure how my personal views on abortion impacted my decision
- What were your reason(s) for wanting an abortion? Select all that apply.
  - I could not afford a baby
  - It would interfere with my education
  - I wanted to focus my attention/resources on children I already had
  - I wanted to focus my resources/attention on my career
  - I did not want to be a single parent
  - I was having relationship problems
  - I was not ready to have a[nother] child
  - Other (Please tell us)
- What would you say was your MAIN reason for wanting an abortion?
  - I could not afford a baby
  - It would interfere with my education
  - I wanted to focus my attention/resources on children I already had
  - I wanted to focus my resources/attention on my career
  - I did not want to be a single parent
  - I was having relationship problems
  - I was not ready to have a[nother] child
  - Other (Please tell us)
- [If during pandemic]: Did the pandemic influence your decision about wanting an abortion in any way?
  - Yes
  - No
  - Unsure
  - I don’t remember

[If yes] Please tell us how the pandemic influenced your decision about wanting an abortion:

**The next set of questions are about your most recent experience trying to get an abortion**

- Which of the following, if any, made it difficult to get an abortion? Please select all that apply.
  - Cost of an abortion
  - Not having insurance coverage for abortion
  - Misinformation about abortion services
  - Lack of information about abortion services
  - Getting to an abortion facility/difficulty with transportation
  - Having to take time off work
  - Having to make childcare arrangements for existing children
  - My partner was not emotionally supportive of my attempt to get an abortion
  - I had no or very few friends or family members that were emotionally supportive of my attempt to get an abortion
  - I did not speak, read, or write in English fluently
  - Too far along in my pregnancy
  - Covid-related clinic closures and/or limited appointment times
  - Other (Please tell us)
  - It was not difficult at all to get an abortion

[*If lack of info*] What type of abortion-related information did you need but couldn’t access? Please select all that apply.

- Where to get an abortion
- Cost of an abortion
- Insurance coverage of abortion
- Information on different abortion procedures
- State laws that could impact your ability to have an abortion
- Other (please tell us)
- Did any of the following factors make it easier to get an abortion? Please select all that apply.
  - I had a financially supportive partner, friend, or family member
  - I had an emotionally supportive partner, friend, or family member
  - My insurance covered the cost of the abortion
  - I was able to use telehealth for abortion counseling or for consenting to abortion
  - I had the option of taking the abortion pills at home
  - I got financial assistance from an abortion fund
  - I could find accurate information about abortion in my native language
  - Other (please tell us)
  - None of the above made it easier for me to get an abortion
- Did you end up getting an abortion?
  - Yes
  - No

[If no] What would you say was the MAIN reason you did not get an abortion for that pregnancy? Select only one:

- - - It was too expensive/I did not have insurance coverage
    - Covid-related clinic closures and/or limited appointment times
    - Misinformation about abortion
    - A lack of information about abortion services
    - Couldn’t take time off work
    - Couldn’t get childcare
    - Lack of transportation
    - I did not speak, read, or write in English fluently
    - Too far along in my pregnancy
    - Changed my mind
    - I miscarried before I could get an abortion
    - Other (Please tell us)

[If yes]

- What type of abortion did you have?
  - Medication (pills)
  - Surgical
  - Other (please tell us)
- Where did you get your abortion?
  - Private doctor’s office
  - Community health or family planning clinic like Planned Parenthood
  - Hospital
  - At home (abortion pills were mailed to me or I picked them up at a pharmacy)
  - Some other place or person (please tell us):
- In which state did you have the abortion?
- How many weeks pregnant were you when you got the abortion?
- *[If during pandemic]* Did the pandemic affect your ability to get an abortion in any way (either positively or negatively)?
  - Yes
  - No
  - Not sure

[If yes]: *Please tell us how the pandemic impacted your ability to get an abortion

- Is there any information or advice you would give to other foreign-born people seeking abortion?
- Is there anything else about your most recent abortion experience you’d like to tell us?

**Below are a few questions about your background that will help us understand how your experiences compare to other people of various backgrounds.**

- How long have you lived in the US? [drop down 0-50 yrs]
- What is the highest level of school that you have completed?
- No formal education
- Less than high school
- Some high school, no diploma
- High school graduate
- Some college, no degree
- Associate degree
- Bachelor degree
- Some advanced schooling (master’s, professional, or doctorate), no degree
- Advanced degree (master’s, professional, or doctorate)
- Prefer not to answer
- What best describes your current gender identity? (Select all that apply)
- Woman
- Man
- Non-binary
- Genderqueer
- Two-Spirit (feel free to include your tribe’s specific language for your identity, if you would like) *[textbox]*
- Additional gender category, please specify: *[textbox]*
- Prefer not to answer
- Do you identify as transgender?
- Yes
- No
- Prefer not to answer
- Do you consider yourself to be (select all that apply)
- Asexual
- Bisexual
- Gay
- Lesbian
- Pansexual
- Queer
- Questioning
- Same-gender loving
- Straight/heterosexual
- Another sexual orientation (please specify)
- Prefer not to answer
- How would you describe your employment and/or student status now? (Select all that apply)
- Working part time
- Working full time
- Student (full time)
- Student (part time)
- Unemployed
- Retired
- Permanently disabled
- Taking care of home or family
- Other (please specify*) [textbox]*
- Prefer not to answer

4.) What is your current relationship status?

- Single (never married)
- In a relationship, not living with partner
- In a relationship, living with partner
- Married or in a civil union
- Divorced or separated
- Widowed
- Other
- Prefer not to answer

5.) Are you of Spanish, Hispanic, or Latin descent?

- Yes
- No
- Prefer not to answer

6.) How would you describe your race? Please mark all that apply.

- Asian or Pacific Islander
- Black/ African American
- Native American/Alaska Native
- White/Caucasian
- Other (please tell us)
- Prefer not to answer

7.) How many times have you been pregnant?

- [Dropdown menu 1, 2, 3, 4, 5+, prefer not to answer]

8.) How many children are you currently parenting? (your own (via birth or adoption), or a partner’s)

- [Numerical text box]
- Prefer not to answer

11.) Do you have health insurance?

- Yes
- No
- I don’t know
- Prefer not to answer

[If yes] What kind of health insurance do you have? Select all that apply.

- Health insurance through your or someone else’s employer or union
- Medicaid or any other state medical assistance plan for people with lower incomes
- TRICARE, a government plan that pays health care bills for active-duty military personnel and their dependents
- Health insurance that you or your family bought directly (e.g., from a state exchange, Obamacare)
- Health insurance from some other source (Please specify): [textbox]
- I do not know
- Prefer not to answer
- The following are challenges some people have experienced when trying to enroll in a health insurance plan. Please select all that you have experienced, if any.
  - I was afraid to enroll in a health insurance plan
  - I didn’t know where to find out about health insurance plans
  - I experienced difficulty enrolling in a plan or finding information because I did not speak, read, or write in English fluently
  - I was confused about whether I was able to enroll a health insurance plan
  - I was confused by the steps in the enrollment process
  - I suddenly lost my health insurance and didn’t understand why
  - I **did not** experience any of the above
  - Prefer not to answer

[If afraid] Please tell us more about why you were afraid to enroll in a health insurance plan.

12.) During the past month, would you say you had enough money to meet your basic living needs such as food, housing, and transportation?

- All the time
- Most of the time
- Some of the time
- Rarely
- Never
- I do not know
- Prefer not to answer
- Which birth control method(s), if any, are you currently using (select all that apply)?
- None—I am not using any method
- Withdrawal (pulling out)
- Birth control pills
- Vaginal ring (NuvaRing, Annovera)
- Shots or injections (Depo-Provera or “Depo”)
- Patch (OrthoEvra patch, Xulane)
- Hormonal IUD (Mirena, Skyla, Liletta, Kyleena)
- Copper IUD (Paragard)
- Implant (Implanon/Nexplanon)
- External condoms (sometimes called male condoms)
- Internal condoms (FC2) (sometimes called female condoms)
- Vaginal barrier methods (diaphragm, sponge)
- Spermicide (cream, gel, film, foam, suppositories)
- Fertility awareness methods (rhythm method, cycle beads, periodic abstinence)
- Tubal ligation (sterilization)
- Partner's vasectomy
- Emergency contraception (also called “EC”, “morning after” pill, Plan B, Ella, Next Choice, EContra, EZ, May Way, After Pill)
- Another method (please tell us) *[textbox]*
- Prefer not to answer

13.) This question is about immigration status. We completely understand if you prefer not to answer this question. If you do, the information will contribute to abortion research, since there is currently no information about abortion access based on a person’s immigration status.

- I’m a US-citizen
- I’m a green card holder
- I have a nonimmigrant visa
- I have an immigration visa
- I’m a DACA recipient
- None of the above
- Prefer not to answer

Thank you for completing this survey about your abortion seeking experience! In addition to the $10 you’ll receive for participating in this study, we’d also like to offer you a free resource guide listing national and local health-related services. A list of local resources are available for the following cities: Atlanta, Boston, Chicago, Los Angeles, New York, San Francisco, and St. Paul and Minneapolis. These guides are available on the study website: <https://bit.ly/abortion_access>

**-------------------------------------------------------------------------------------------------------------------**

**Background Info**

- ¿Cuántas veces **ha intentado obtener** un aborto mientras vivía en los Estados Unidos, incluyendo las veces que no logró obtener un aborto? [Drop-down 1-10+]
- ¿Cuántos abortos ha obtenido en los Estados Unidos? [Drop down 0-5+]
  - [If not 0] ¿En qué estado(s) pudo conseguir un aborto? Seleccione todos que correspondan [Drop down states]

**Para las siguientes preguntas, por favor piense en la última vez que intentó acceder a servicios de aborto.**

- ¿Cuántas semanas tenía de embarazada/e/o cuando se dio cuenta de que estaba embarazada/e/o?
- ¿En qué estado estaba viviendo en ese momento? [drop down]
- ¿Intentó obtener un aborto después del 13 de marzo 2020 (desde el inicio de la pandemia)?
  - Sí
  - No
- Una vez que supo que estaba embarazada/e/o, ¿sabía inmediatamente que quería abortar?
  - Sí
  - No
  - No recuerdo
- Una vez que supo que estaba embarazada/e/o, ¿qué hizo?
  - Intenté conseguir más información sobre el aborto
  - Hablé con alguien o intenté contactar a alguien
  - Hice una cita
  - Tomé tiempo para pensarlo
  - Otro (por favor cuéntenos):
  - No recuerdo

[If online research] Por favor, cuéntenos lo que buscaba:

[If talked to someone AND didn’t know right away OR Unsure] ¿Con quién quería hablar primero cuando estaba tratando de decidir si el aborto era la decisión indicada para usted?

- - - - Pareja
      - Familia
      - Amiga/e/o
      - Profesional de salud
      - Otra persona

[If provider] Por favor, cuéntenos ¿con qué tipo de profesional de salud habló? (Por ejemplo, ¿habló con un/a/e proveedor de aborto? ¿Un/e/a médico/e/a de familia? ¿una/e/o farmacéutica/e/o?) (open-ended)

[If talked to someone AND didn’t know right away OR unsure] ¿Habló con alguna otra persona mientras trataba de decidir si quería obtener un aborto?

- - - - Sí 🡪 Por favor, cuéntenos:
      - No
      - No recuerdo

[If talked to someone AND didn’t konw right away OR unsure AND during pandemic]: ¿Las medidas de distanciamiento social o las recomendaciones por la pandemia influyeron con quién habló sobre el aborto?

- - - - Sí
      - No
      - No sé

[If talked to someone and knew right away] ¿Con quién habló primero sobre su decisión de abortar?

- - - - Pareja
      - Familia
      - Amiga/e/o
      - Profesional de salud
      - Otra persona

[If provider] Por favor, cuéntenos ¿con qué tipo de profesional de salud habló? (Por ejemplo, ¿habló con un/a/e proveedor de aborto? ¿Un/e/a médico/e/a de famlia? ¿una/e/o farmacéutica/e/o?) (open-ended)

[If talked to someone AND knew right away] ¿Habló con alguna otra persona mientras trataba de decidir si quería obtener un aborto?

- - - - Sí 🡪 Por favor, cuéntenos:
      - No
      - No recuerdo

[If talked to someone AND knew right away AND during pandemic]: ]: ¿Las medidas de distanciamiento social o las recomendaciones por la pandemia influyeron con quién habló sobre el aborto?

- - - - Sí
      - No
      - No sé

[If talked to someone] ¿La opinión de alguien le hizo sentir más segura/e/o en abortar?

- - - - Sí
      - No
      - No estoy segura/e/o
      - No recuerdo

[If talked to someone] ¿La opinión de alguien le hizo ya no querer abortar?

- - - - Sí
      - No
      - No estoy segura/e/o
      - No recuerdo
- ¿Cómo impactaron sus puntos de vista personales sobre el aborto su decisión de intentar de abortar?
  - Hicieron muy fácil la decisión
  - Hicieron mas o menos fácil la decisión
  - Hicieron algo difícil la decisión
  - Hicieron muy difícil la decisión
  - Mis puntos de vista personales no impactaron a mi decisión
  - No estoy segura/e/o de como mis puntas de vista personales sobre el aborto impactaron a mi decisión
- ¿Cuáles eran las razones principales por las cuales quería abortar? Seleccione todas las que correspondan
  - No tenía soporte económico para tener un bebe
  - Iba a interferir con mi educación
  - Quería enfocar mi atención y/o recursos a les niñes que ya tenía
  - Quería enfocar mi atención / recursos en mi carrera
  - No quería ser madre/xadre/padre soltera/e/o
  - Estaba teniendo problemas con mi pareja
  - No estaba lista/e/o para tener un (u otro) niño
  - Otro (Por favor, cuéntanos):
- ¿Qué diría que era **su razón principal** por querer abortar?
  - No tenía soporte económico para tener un bebe
  - Iba a interferir con mi educación
  - Quería enfocar mi atención y/o recursos a les niñes que ya tenía
  - Quería enfocar mi atención / recursos en mi carrera
  - No quería ser madre/xadre/padre soltera/e/o
  - Estaba teniendo problemas con mi pareja
  - No estaba lista/e/o para tener un (u otro) niño
  - Otro (Por favor, cuéntanos):
- [If during pandemic]: ¿La pandemia influyó su decisión sobre querer abortar de alguna forma?
  - Sí
  - No
  - No estoy segura/e/o
  - No recuerdo

[If yes]: Por favor cuéntenos cómo la pandemia influyó a su decisión de querer abortar:

**Las siguientes preguntas son para entender más sobre su última experiencia tratando de obtener un aborto.**

- ¿Cuál de las siguientes le hizo difícil obtener un aborto? Por favor, seleccione todas las que correspondan
  - El costo
  - No tener cobertura del seguro para el aborto
  - Desinformación sobre los servicios del aborto
  - Falta de información sobre los servicios del aborto
  - Llegar a un centro de aborto/ dificultad con el transporte
  - Tener que faltar al trabajo
  - Tener que arreglar para que alguien cuidara a mis niñes
  - Mi pareja no me apoyaba emocionalmente en mi intento a abortar
  - No tenía o tenía muy pocos/as/es amigo/as/es o familia que apoyaba emocionalmente a mi decisión
  - No hablaba, leía, o escribía inglés con fluidez
  - Estaba demasiado avanzada/e/o en el embarazo
  - Cierres de clínicas o horarios de citas limitados por covid-19
  - Otro (por favor, cuéntenos):
  - No fue difícil obtener un aborto

[If lack of info] ¿Qué tipo de información relacionada al aborto necesitaba pero no podía acceder? Seleccione todo que corresponda

- - Donde acceder a servicios de aborto
  - Precio del aborto
  - Cobertura del seguro del aborto
  - Información sobre diferentes procedimientos del aborto
  - Leyes estatales que podrían impactar a su capacidad de tener un aborto seguro
  - Otro (por favor, cuéntanos):
- ¿Alguno de los siguientes factores hizo que fuera más fácil obtener un aborto? Por favor, seleccione todos los que correspondan.
  - Tenía una pareja, amiga/e/o o familia que me apoyaba económicamente
  - Tenía una pareja, amiga/e/o o familia que me apoyaba emocionalmente
  - Mi seguro cubrió el costo del aborto
  - Pude usar Telehealth para el asesoramiento del aborto o para dar consentimiento al aborto
  - Tenía la opción de tomar los medicamentos del aborto en casa
  - Pude encontrar la información precisa sobre el aborto en mi lengua materna
  - Otro (por favor, cuéntenos):
  - Ninguno de estos factores hizo más fácil obtener un aborto
- ¿Pudo abortar?
  - Sí
  - No

[If no] ¿Qué diría que era **la razón principal** por la cual no obtuvo un aborto por ese embarazo? Seleccione solo uno:

- - Fue demasiado caro/No tenía seguro
  - Cierres de clínicas o horarios de citas limitados por covid-19
  - Desinformación sobre el aborto
  - Falta de información sobre los servicios del aborto
  - No podía faltar al trabajo
  - No podía arreglar para que alguien cuidara a mis niñes
  - Faltaba transporte
  - No hablaba, leía, o escribía inglés con fluidez
  - Estaba demasiado avanzada/e/o en el embarazo
  - Cambié mi mente
  - Tuve un aborto espontáneo antes de que podía abortar
  - Otro (por favor, cuéntenos):

[If yes]

- ¿Qué tipo de aborto tuvo?
  - Con medicamentos (píldoras)
  - Quirúrgico
  - Otro (por favor, cuéntenos):
- ¿Dónde consiguió su aborto?
  - Consultorio médico privado
  - Una clínica de la comunidad o de planificación familiar como Planned Parenthood
  - En un hospital
  - En casa (me mandaron los medicamentos del aborto o los recogí en la farmacia)
  - Otro lugar/ persona (por favor, cuéntenos):
- ¿En qué estado obtuvo su aborto?
- ¿Cuántas semanas tenía de embarazada/e/o cuando obtuvo el aborto?
- [if during pandemic] ¿La pandemia afectó a su capacidad de obtener el aborto de alguna manera (positivamente o negativamente)?
  - Sí
  - No
  - No estoy segura/e/o

[If yes]: *Por favor cuéntenos como la pandemia impactó a su capacidad de obtener un aborto

- ¿Hay información o consejos que daría a otras personas nacidas al extranjero buscando obtener un aborto?
- ¿Hay algo más sobre su última experiencia del aborto que le gustaría contarnos?

**Abajo hay algunas preguntas sobre sus antecedentes que nos ayudarán a entender como su experiencias comparan a otras personas de orígenes diversos.**

- ¿Por cuánto tiempo ha vivido en los Estados Unidos? [drop down 0-50 years]
- ¿Cuál es el nivel más alto de la escuela que ha completado?
  - Educación no formal
  - Soy graduada/e/o de primaria
  - Alguna escuela secundaria, sin diploma
  - Soy graduada/e/o de escuela secundaria
  - Algunos estudios universitarios, sin título
  - Associate’s Degree
  - Obtuve bachillerato
  - Algunos estudios avanzados (maestría, profesional o doctorado), sin título
  - Título avanzado (maestría, profesional o doctorado)
  - Prefiero no responder
- ¿Cuál de las siguientes opciones mejor describe su identidad de género actual? Seleccione todas las que correspondan
  - Mujer
  - Hombre
  - No-binario
  - Genderqueer
  - Two-spirit (siéntese libre de incluir el lenguaje especifico de su tribu si quiere) [textbox]
  - Categoría de género adicional, por favor especifique: [text box]
  - Prefiero no responder
- ¿Se identifica como transgénero?
  - Sí
  - No
  - Prefiero no responder
- ¿Usted se considera…? Seleccione todas las que correspondan
  - Asexual
  - Bisexual
  - Gay/homosexual
  - Lesbiana/e/o
  - Pansexual
  - Queer
  - Cuestionándome
  - Amante del mismo género
  - Straight/heterosexual
  - Otra orientación sexual (por favor, especifique):
  - Prefiero no responder
- ¿Cómo describiría su estado de empleo y/o estado estudiantil ahora? Seleccione todas las que correspondan:
  - Trabajando medio tiempo
  - Trabajando a tiempo completo
  - Estudiante (tiempo completo)
  - Estudiante (tiempo parcial)
  - Desempleada/e/o
  - Jubilada/e/o
  - Permanentemente discapacitada/e/o
  - Cuidando a la casa o familia
  - Otro (por favor, especifique) [text box]
  - Prefiero no responder
- ¿Cuál es su estado actual de relación?
  - Soltera/e/o (nunca casada/e/o)
  - En una relación, no viviendo en pareja.
  - En una relación, viviendo en pareja.
  - Casada/e/o o en una unión civil.
  - Divorciada/e/o o separada/e/o
  - Viuda/e/o
  - Otro
  - Prefiero no responder

5.) ¿Eres de ascendencia española, hispana o latina?

- - Sí
  - No
  - Prefiero no responder

6.) ¿Con que raza(s) se identifica? Por favor marque todo lo que corresponda.

- - Asiática/e/o / de las Islas del Pacífico
  - Negra/e/o / afroamericana/e/o
  - Nativo americana/e/o / nativa/e/o de Alaska
  - blanca/e/o / caucásica/e/o
  - Otro (por favor, cuéntenos)
  - Prefiero no responder
- ¿Cuántas veces ha estado embarazada/e/o?
  - [Drop down 1, 2, 3, 4, 5+, prefiero no responder]
- ¿Cuántes hijes está criando actualmente? (Puede ser hijes biológiques, de adopción o de una pareja)
  - [Numerical text box]
  - Prefiero no responder
- ¿Tiene seguro de salud?
  - Sí
  - No
  - No sé
  - Prefiero no responder

[If yes], ¿Qué tipo de seguridad de salud tiene?:

- - Seguro de salud a través de su empleador o sindicato o el de otra persona
  - Medicaid o cualquier otro plan estatal de asistencia médica para personas con bajos ingresos.
  - TRICARE, un plan del gobierno que paga las facturas de atención médica para el personal militar en servicio activo y sus dependientes
  - Seguro de salud que usted o su familia compraron directamente (por ejemplo, de un intercambio estatal, Obamacare)
  - Seguro de salud de alguna otra fuente (especifique): [Text box]
  - Yo no sé
  - Prefiero no responder
- Las siguientes declaraciones son desafíos que algunas personas han encontrado mientras trataban de inscribirse en un seguro médico. Por favor seleccione todas que ha encontrado usted, si es que ha encontrado alguna dificultad.
  - Tenía miedo de inscribirme en un seguro médico
  - No sabía donde encontrar información sobre los planes de seguro médico
  - Encontraba dificultad inscribirme en un plan o encontrando información porque no hablaba, leía o escribía en inglés con fluidez
  - No sabía si podía inscribirme en un plan de seguro médico
  - Me confundían los pasos del proceso de inscripción
  - De repente perdí mi seguro y no entendía porqué
  - No encontré ninguna de las dificultades enumeradas
  - Prefiero no responder

[If afraid] Por favor, cuéntenos más sobre porqué tenía miedo de inscribirse en un plan de seguro médico.

- Durante el último mes, ¿diría que tiene suficiente dinero para satisfacer sus necesidades básicas de vida, como alimentos, vivienda y transporte?
  - Sí, todo el tiempo
  - La mayoría del tiempo
  - A veces
  - Casi nunca
  - Nunca
  - No sé
  - Prefiero no responder
- ¿Qué método(s) anticonceptivo(s) está usando actualmente, si es que está usando algún método anticonceptivo (seleccione todos que correspondan)?
  - Ninguno- no estoy usando ningún método
  - Método de retiro (a veces llamado “marcha atrás”)
  - Las píldoras anticonceptivas
  - El anillo vaginal (NuvaRing, Annovera)
  - Inyecciones (Depo-Provera o “Depo”)
  - Parche (OrthoEvra patch, Xulane)
  - DIU Hormonal (Mirena, Skyla, Liletta, Kyleena)
  - DIU de cobre IUD (Paragard)
  - Implante (Implanon/Nexplanon)
  - Condones extérnales (a veces llamados condones masculinos)
  - Condones intérnales (FC2) (a veces llamado condones femeninos)
  - Métodos de barrera (diaphragm, sponge)
  - Espermicida (crema, gel, film, espuma, supositorios)
  - Determinación de fertilidad (método de ritmo, cuentas de ciclo, abstinencia periódica)
  - Ligadura de trompas (esterilización)
  - Vasectomía de pareja
  - Contracepción de emergencia (también llamadas “píldora del día después”, Plan B, Ella, Next Choice, EContra, EZ, May Way, After Pill)
  - Otro método (por favor, especifique) *[textbox]*
  - Prefiero no responder
- Esta pregunta es sobre el estado de inmigración. Comprendemos completamente si prefiere no responder. Si responde, la información contribuirá a la investigación del aborto, ya que actualmente no hay información sobre el acceso al aborto seguro en relación con el estado de inmigración de una persona.
  - Soy ciudadana/e/o estadounidense
  - Tengo una tarjeta verde
  - Tengo una visa de no inmigrante
  - Tengo una visa de inmigración
  - Soy destinataria/e/o de DACA
  - Ninguna de los anteriores
  - Prefiero no responder

¡Gracias por completar la encuesta sobre su experiencia buscando obtener servicios de aborto! Además de los $10 que va a recibir por participar en este estudio, también nos gustaría ofrecerle una guía gratuita de recursos que enumera servicios relacionados a la salud al nivel nacional y local. Las listas de recursos locales son disponibles para las siguientes ciudades: Atlanta, Boston, Chicago, Los Angeles, New York, San Francisco, y St. Paul y Minneapolis. Estas guías están disponibles en el sitio del estudio: https://bit.ly/acseso-al-aborto
