# Supplementary material for: Recruiting foreign-born individuals who have sought an abortion in the United States: Lessons from a feasibility study
Source: Front Glob Womens Health. 2023 Apr 18;4:1114820. doi: 10.3389/fgwh.2023.1114820 (PMC10151930; doi:10.3389/fgwh.2023.1114820)
Supplement: Supplementary file 1 [file Datasheet1.zip › Appendix 7.DOCX]

**Appendix 7 – mTurk Screening Questionnaire**

**Screening Questions (only taken by Amazon mTurk workers who identify as female, located in US, HIT approval rate (not rejected) 90%)**

1. What country were you born in? [drop down list of countries]

- If US is selected ->[ineligible]

2. Have you ever tried to get an abortion in the United States, even if you didn’t end up getting one?

- - Yes, in the past 2 years
  - Yes, over 2 years ago [ ineligible]
  - Yes, I’m currently trying get an abortion [ineligible]
  - No [Ineligible]

3. How old are you? [Drop down 18-60]

- if>49, ineligible

4. Are you interested in taking a 20 min online survey about your abortion experience? We will compensate you $10 for completing the survey.

- - Yes
  - No

[If yes] The survey is available in English and Spanish. Which language would you prefer to take it in?

- English
- Spanish

--------------------------------------------------------------------------------------------------------------------------------------

1. ¿En qué país nació? [lista desplegable de países]

- Si se selecciona EE. UU. -> [ineligible]

2. ¿Alguna vez ha intentado obtener un aborto en los Estados Unidos?

- Sí, en los últimos 2 años.
- Sí, hace más de 2 años [ ineligible]
- Sí, actualmente estoy intentando abortar [ileligible]
- No [ ineligible]

3. ¿Cuántos años tiene? [Desplegar 18-60]

- If >49, ineligible

4. ¿Le interesa tomar una encuesta en línea de 20 minutos sobre su experiencia con el aborto? Le daremos $10 de compensación por completar la encuesta.

- Sí
- No

[If yes] La encuesta se puede hacer en inglés y español. ¿En qué idioma prefiere tomar la encuesta?

- inglés
- español
